# Supplementary material for: Elastic analysis bridges structure and dynamics of an AAA+ molecular motor
Source: PLoS Comput Biol. 2025 Oct 24;21(10):e1013596. doi: 10.1371/journal.pcbi.1013596 (PMC12582506; doi:10.1371/journal.pcbi.1013596)
Supplement: S1 Text — (PDF) [file pcbi.1013596.s001.pdf]

Supplementary information for: Elastic analysis bridges structure and dynamics of an AAA+ molecular motor

Victor Hugo Mello
Gulbenkian Institute for Molecular Medicine (GIMM), Lisbon, Portugal

Jiri Wald
University Medical Center Hamburg-Eppendorf (UKE),
Institute of Microbial and Molecular Sciences, Hamburg, Germany
Centre for Structural Systems Biology (CSSB), Hamburg, Germany and
Deutsches Elektronen-Synchrotron Zentrum (DESY), Hamburg, Germany

Thomas C Marlovits
University Medical Center Hamburg-Eppendorf (UKE),
Institute of Microbial and Molecular Sciences, Hamburg, Germany
Centre for Structural Systems Biology (CSSB), Hamburg, Germany and
Deutsches Elektronen-Synchrotron Zentrum (DESY), Hamburg, Germany

Pablo Sartori*
Gulbenkian Institute for Molecular Medicine (GIMM), Lisbon, Portugal and
**pablo.sartori@gimm.pt*

APPENDIX A: COMPARISON BETWEEN ELASTIC PSEUDOENERGY AND OTHER METRICS

In order to assess the relevance of the elastic pseudoenergy introduced in this work, we compared it against a diverse set of established metrics that have been used to characterise protein energetics, deformation, and flexibility. While each of these reference metrics is grounded in different frameworks and is associated with different spatial scales, they all serve as proxies for understanding the energetic and structural behaviour of proteins.

The first comparison involves **biophysics-based energy estimations**, where proteins are treated as systems transitioning between discrete functional states — such as active and inactive conformations — guided by free energy differences. These models, often parameterised from experimental data, provide a simplified yet informative representation of protein dynamics and energetics.

Second, we consider **force-field-based energy estimations**, which form the basis of molecular dynamics (MD) simulations. In this framework, atomic or coarse-grained interactions are encoded in empirical potential functions known as force fields, which control the time evolution of protein structures under thermal fluctuations. These simulations produce energetically informed trajectories that reflect the dynamic behaviour of proteins *in silico*.

We also explore metrics based on **Ramachandran angles**, which offer a geometric and energetic perspective on protein backbone conformation. Deviations from the well-characterised (ϕ, ψ) angle distributions – originally established by Ramachandran and colleagues [1] – are commonly interpreted as indicators of structural strain or steric hindrance.

Finally, we examined correlations with the ***β*-factor**, a metric routinely derived from X-ray crystallography and cryo-electron microscopy data. Although originally introduced to quantify atomic positional uncertainty, β-factors have been implicated in reflecting aspects of conformational entropy, local flexibility, and even functional motion, thus making them a valuable, yet indirect, probe of intrinsic structural variability [2–4].

By comparing the elastic pseudoenergy to these four distinct metrics, we aim to test whether our formulation correlates with biophysical and structural features already encoded in well-established energetic and flexibility measures.

Biophysics-based energy estimation

While it would be desirable to study RuvB within this framework to compare with our estimates of elastic pseudoenergy, several challenges arise. As we show in the main text, RuvB function involves coordination among subunits that themselves can adopt numerous conformations, making such energy inference difficult. Instead, we applied this approach to a cooperative model of haemoglobin conformational changes, a classical system for studying allostery and cooperativity at equilibrium that has been investigated for over a century [5].

Among the extensive literature on oxygen binding at haemoglobin's four heme-containing sites [6], a notable model is the Monod-Wyman-Changeux (MWC) model, which introduced the causal relationship between conformational changes and cooperativity [7]. Also, this model was the first to refer to distinct conformational states of the haemoglobin tetramer, T (tense) and R (relaxed), with different affinities for binding oxygen. The nomenclature remained established in the field after experimental verification of distinct conformational states. We collected examples for different types of models for the O_2 saturation curves of haemoglobin and show in Table I.

**Model type**	**Reference**	**Energy difference (kcal/mol)**
Adair's model	Imai, 1982 [8]	1.3 - 3.6*
Molecular code	Smith & Ackers, 1985 [9]	5.9 $\pm$ 0.2
Molecular code	Parrella et al., 1990 [10]	7
Molecular code	Huang et al., 1996 [11]	6.3 $\pm$ 0.2
Concerted (MWC)	Yonetani et al., 2002 [12]	0.7 - 3.1*
Elastic pseudoenergy	This study ($\alpha_2\beta_2$)	6.7 $\pm$ 0.5
Elastic pseudoenergy	This study (2 $\alpha\beta$)	5.2 $\pm$ 0.4

TABLE I. *Energy difference between haemoglobin R and T states from biophysical models and elastic pseudoenergy.* The values for estimated energy difference were derived from fitting experimental data to cooperative models in various experimental approaches. *Estimates of free-energy obtained from experiments in different buffer conditions.

Taking advantage of multiple structures of haemoglobin assemblies solved at T and R states [13], we computed the pairwise elastic pseudoenergy using the same approach we applied for the RuvB analysis (check the Supplementary methods for further details). To be precise, the dataset we used consists of crystallographic structures in the fully oxygenated state, or R (PDBs: 1LFQ, 1LFT, 1LFV, and 1LFY) and in the fully deoxygenated state, or T (PDB: 1LFL). The R structures display a single $\alpha\beta$ pair per unit cell, whereas the T structure has two full tetramers per unit cell. With these structures, we performed two types of pairwise comparisons: first, considering $\alpha\beta$ chains, resulting in four R states and four T states (Fig 1A); and second, considering the full tetramers $\alpha_2\beta_2$, resulting in four R states (applying symmetry operations) and two T states (Fig 1B). Using this approach, we calculated the elastic pseudoenergy difference of R x R pairs, T x T pairs, and T x R pairs. The advantage of using both approaches is that we can account (or not) for elastic deformations in the interface between two $\alpha\beta$ subunits. We also included the mean $\pm$ standard deviation for each group in Table I in units of kcal/mol to facilitate the comparison with the other observations.

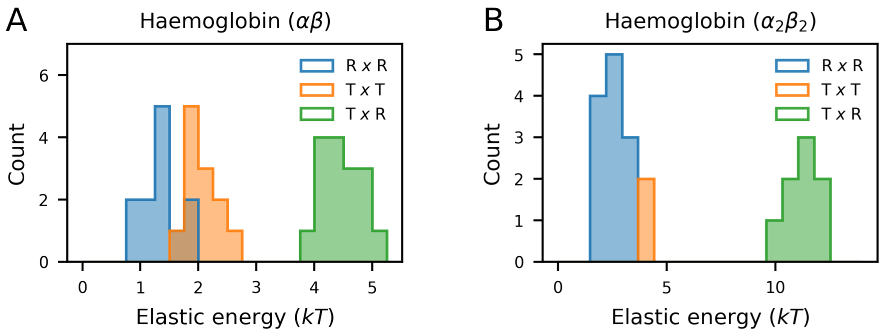

FIG. 1. *Haemoglobin elastic pseudoenergy difference from pairwise comparisons.* **A.** Comparison of individual $\alpha\beta$ pairs. With four pairs within each group, there is a total of 16 pairwise comparisons for R x R, T x T, and T x R. **B.** Comparison of $\alpha_2\beta_2$ tetramers. With four R and two T tetramers, the number of comparisons is 16 for R x R, 2 for T x T, and 8 for T x R.

Analysing the magnitude of elastic pseudoenergy in the dataset we used, we can pose interesting observations regarding haemoglobin conformational changes:

- Our results for T x R comparisons are in the same order of magnitude as those obtained with different methods and probed using very different experimental approaches. Besides, our estimate of elastic pseudoenergy falls within the equivalence range for the molecular code model. However, instead of supporting the validity of a specific model, we would rather interpret that our energy estimates match the order of magnitude of multiple biophysical models. Using strain analysis to corroborate a specific biophysical model of oxygen binding with haemoglobin requires a dedicated study for this purpose.

- Our observations reveal a consistent ordering of elastic pseudoenergy difference between types of comparison, namely, R x R < T x T < T x R. This suggests that (*a*) the energy difference between T and R states is significantly larger than those observed for T and R individually; (*b*) the R states are less variable than T states, which is in remarkable agreement with the analysis performed by the authors who solved these structures [13]. While the first observation comes with no surprise, the second is counterintuitive given the labels Relaxed and Tense. This suggests that having oxygen bound to haemoglobin has a stabilising effect on its structure.
- Comparing the same groups using only two or all four chains provides an interesting pattern: whereas R x R and T x T comparisons with $\alpha\beta$ have exactly half of the elastic pseudoenergy of $\alpha_2\beta_2$, that is not the case for T x R comparisons. This is illustrated in the last two items of Table I. This result implies that there is a significant signal of deformation localised in the interface between the two $\alpha\beta$ dimers exclusively in R $\leftrightarrows$ T transitions. This particular observation is known since 1968, when the first structures of oxyhaemoglobin and deoxyhaemoglobin were compared [14].

In summary, here we present a system where both biophysical measures of cooperative energy and structural information were available for a pair of conformational states. Using protein elasticity, we find that the elastic pseudoenergy difference between these states corresponds to the free-energy difference derived from biophysical models, within the same order of magnitude.

Force-field-based energy estimation

To examine how the elastic pseudoenergy calculated in this study relates to force-field-based energy estimates, we selected the all-atom force field model ff19SB [15], which is currently recommended by Amber [16], a widely used suite of programs in the molecular dynamics community. Our objective was to use this force field solely to quantify the energy of different RuvB conformations.

The protocol we followed is outlined as follows: *(i)* We converted PDB files of monomeric RuvB conformations into an Amber-compatible format using `pdb4amber`, ensuring that only residues present in all conformations were retained. *(ii)* Using the program `tleap`, we generated a simulation environment for each structure individually. We selected an implicit solvent model to prevent the initial coordinates of water molecules from influencing the energy calculations. *(iii)* We employed the program `sander` to simulate a trajectory consisting of a single iteration step. To preserve the original atomic coordinates, we deliberately avoided energy minimization, a procedure commonly used in MD protocols.

Unlike protein strain analysis, force-field calculations do not rely on a reference structure as a baseline. Consequently, we compared the elastic pseudoenergy to both the absolute value of the elastic pseudoenergy for each conformation in the cycle and the energy difference between cycle and reference conformations (see Fig 2). We observed a significant correlation only for the energy difference, which was negative. This negative yet statistically significant correlation may be a consequence of the reference structure we chose. Because s_0 requires an additional ATP hydrolysis step to start the mechanochemical cycle, it might have an excess of free-energy compared to the reference structures.

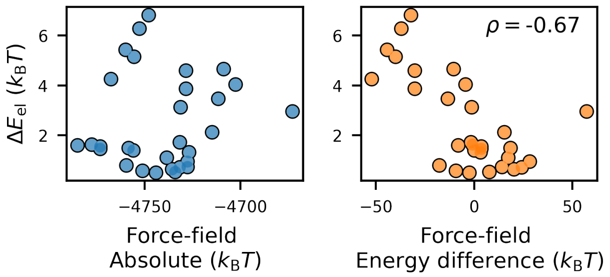

FIG. 2. *Correlation of elastic pseudoenergy and force-field derived energies.* Scatter plots show the energy values obtained for the cycle conformations. The total elastic pseudoenergy calculated from strain analysis is shown on the vertical axis, and the Amber force-field ff19SB energies on the horizontal axis. The left panel represents the absolute energy of each monomeric conformation, whereas the right panel represents the energy difference between the cycle and its corresponding reference. The strain-based elastic pseudoenergy correlates significantly with the force-field energy difference between cycle and reference ($p = 5 \cdot 10^{-5}$).

Additionally, the total energy amplitudes obtained from force-field calculations exceeded $100 k_B T$, which would correspond to an elastic model with a stiffness constant more than ten times greater than typical estimates for protein

stiffness. However, this high amplitude far surpasses the non-equilibrium energy input from the ATP hydrolysis cycle and, therefore, is not consistent with the behaviour expected of a processive motor.

Ramachandran angles

Due to the striking conservation of Ramachandran angle distributions across amino acids of the same type, the fraction of outliers is widely used as a criterion for assessing the quality of atomistic protein models [17]. Because of such relationship between conformation and energetics, we compared our inferred elastic pseudoenergy to two metrics: the Ramachandran score calculated using a standard validation tool; and the variation in Ramachandran angles between cycle and reference structures.

For the first comparison, we employed MolProbity (updated January 2024) [17], a PDB validation tool, to calculate the Ramachandran score (here denoted as s) for each residue. This score, derived from dihedral angles, ranks residues on a scale from 0% to 100% based on the angular distributions observed in curated high-quality structures. Lower s values correspond to less populated regions of the distribution. To ensure parity with our structural comparison approach, we assigned each residue the minimum s-value between the cycle and reference structures.

Residues were grouped into bins ($s \leq 1\%$, $1\% < s \leq 2\%$, $2\% < s \leq 4\%$, $4\% < s \leq 10\%$, $s > 10\%$) for each amino acid type (Fig 3). Despite limited sample sizes in lower s bins, we observed a trend of increasing median elastic pseudoenergy as s decreased. This suggests a detectable association between high backbone torsion and elevated elastic pseudoenergy. Glycine residues, however, deviated from this pattern, exhibiting lower elastic pseudoenergy at intermediate s values. This anomaly likely stems from glycine's exceptional flexibility, due to its minimal steric hindrance (its side chain consists of a single hydrogen atom).

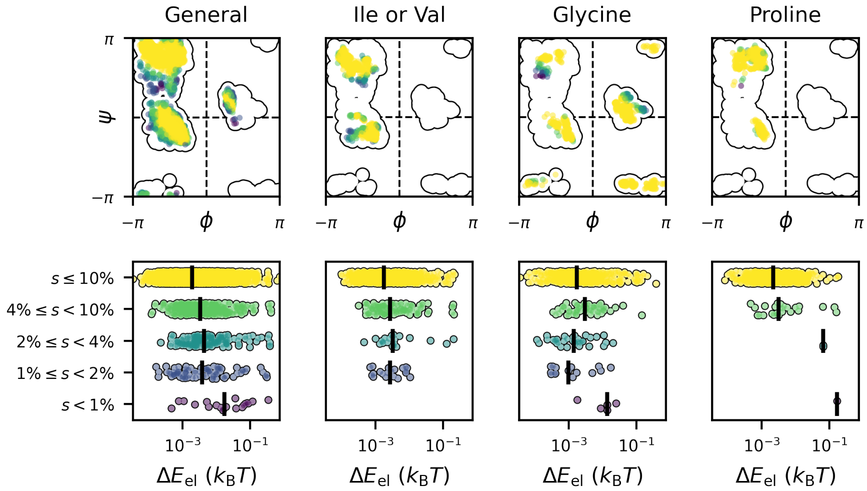

FIG. 3. *Residues near forbidden areas of Ramachandran plot tend to have higher elastic pseudoenergy.* On top, the backbone dihedral angles ϕ and ψ are shown in the Ramachandran scatter plot colored by groups from yellow (far from forbidden regions) to purple (close to forbidden regions). Each panel represents a category of residues with specific steric constraints due to their side chain chemical composition. The contour lines draw the boundaries containing all RuvB residues in all conformations. Below, residues belonging to the corresponding category above are binned in groups according to their Ramachandran score measured with MolProbity. Black dashes mark the median of the distribution for each bin.

We next examined correlations between elastic pseudoenergy and changes in Ramachandran angles. For each residue, we computed $\Delta\phi^2 = \left(\phi_{\text{cycle}} - \phi_{\text{ref}}\right)^2$ and $\Delta\psi^2 = \left(\psi_{\text{cycle}} - \psi_{\text{ref}}\right)^2$ to quantify angular shifts.While both elastic pseudoenergy and dihedral angle differences serve as local deformation estimates, they operate at distinct spatial scales. Dihedral angles depend on the coordinates of four covalently linked backbone atoms, making them sensitive to highly localised conformational changes. In contrast, elastic pseudoenergy aggregates atomic displacements within a 9 Å radius, capturing deformations across residues that may not be adjacent in sequence.

Significant correlations emerged between elastic pseudoenergy and Ramachandran angle variations at both residue and conformation levels, particularly in their first and second moments (Fig 4). Notably, correlations at the whole-conformation scale were far stronger than those at the residue level. This discrepancy implies that while individual residues may exhibit weak associations between deformation metrics, non-local compensatory effects in backbone

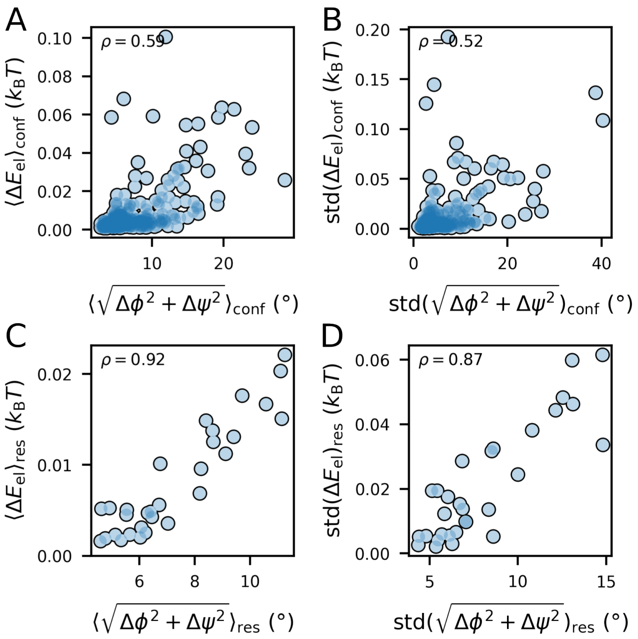

FIG. 4. *Correlations between elastic pseudoenergy and Ramachandran angle variation.* **A.** Comparison of means across conformations (correlation test, $p = 4 \cdot 10^{-30}$). **B.** Comparison of standard deviation across conformations ($p = 8 \cdot 10^{-23}$). **C.** Comparison of means across residues ($p = 4 \cdot 10^{-13}$). **D.** Comparison of standard deviation across residues ($p = 4 \cdot 10^{-10}$).

torsion collectively produce a coherent deformation profile at the global scale.

β-factor

A comprehensive free-energy description of a protein includes both enthalpic and entropic contributions, which in principle links β-factors to the free-energy landscape of protein structures. However, caution is warranted when comparing β-factors to the elastic pseudoenergy estimates generated by our methodology. The elastic pseudoenergy calculated here does not directly account for entropic effects on atomic positions and thus primarily reflects enthalpic (internal) energy rather than the full free-energy. Moreover, there is no fundamental reason to expect a direct correlation between enthalpic and entropic forces. Additionally, regions of low structural resolution could bias elastic pseudoenergy estimates to larger values, potentially leading to spurious correlations between energy and entropy.

To explore the relationship between elastic pseudoenergy and β-factors, we computed the mean β-factor per residue (averaged across 30 conformations) and per conformation (averaged over all residues), considering the maximum β-factor between cycle and reference structures for each case. Our analysis revealed significant correlations between elastic pseudoenergy and β-factors in both the first and second moments at the residue scale, and in the first moment at the conformation scale (Fig 5). These findings are consistent with previous studies that reported similar correlations between strain-based metrics and β-factors [18, 19]. Importantly, while positive correlations alone cannot distinguish whether elevated elastic energies arise from imprecise atomic coordinates or from regions of high conformational entropy, the collective results from this and previous analyses suggest that the elastic pseudoenergy we calculate is not simply an artifact. Rather, it robustly aligns with multiple energy-related definitions across structural scales.

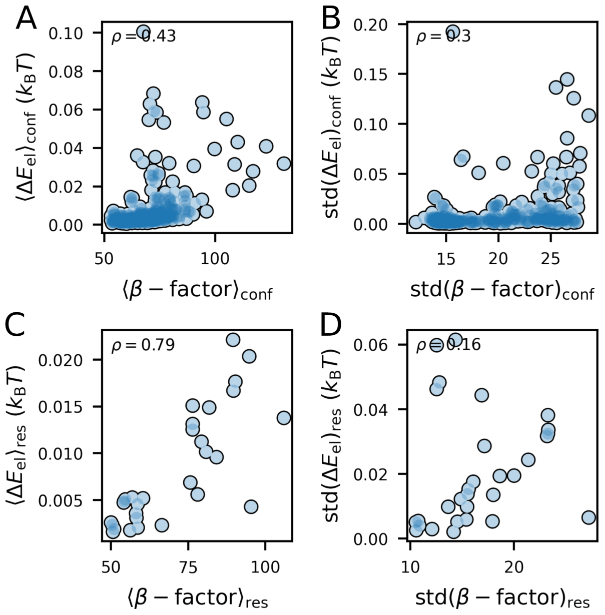

FIG. 5. *Correlations between elastic pseudoenergy and β-factor.* **A.** Comparison of means across conformations (correlation test, $p = 2 \cdot 10^{-15}$). **B.** Comparison of standard deviation across conformations ($p = 8 \cdot 10^{-8}$). **C.** Comparison of means across residues ($p = 2 \cdot 10^{-7}$). **D.** Comparison of standard deviation across residues ($p = 0.4$).

APPENDIX B: ELASTIC MODULI OF PROTEINS

Different elastic moduli are related to various forms of deformation exerted on materials, such as shear (shear modulus), compression (bulk modulus), or one-directional extensions (Young's modulus). Most measurements of the Young modulus of proteins were obtained for polymers (summarised in [20, 21]), which might not reflect the elastic properties of single molecules due to relative displacement between protomers. These estimates range several orders of magnitude, spanning 1MPa to 10GPa. We then decided to base our stiffness constant on studies where the Young modulus was inferred using atomic force microscopy on single molecules of globular proteins. The results range from 2.5-9MPa in [22], 4-50MPa [23], 2-75MPa in [24], and 500MPa in [25]. Using a different nanorheology approach, Wang and Zocchi estimate Young's modulus of a globular protein to be around 11MPa [26].

The elastic pseudoenergy density in the Saint Venant-Kirchhoff model depends linearly on proteins' stiffness in parameters λ and μ, which are themselves functions of the Young modulus and Poisson's ratio. Because the estimates of protein stiffness vary by orders of magnitude, our estimations of elastic pseudoenergy also comprise a large uncertainty in its amplitude. The dependency of λ and μ on Poison's ratio is non-linear and mostly flat for values close to $\frac{1}{3}$, typically assumed for proteins [25]. For our analysis, we considered $Y = 100\text{MPa}$ and $\nu = \frac{1}{3}$, which led respectively to $\lambda \approx 73\text{MPa}$ and $\mu \approx 38\text{MPa}$.

APPENDIX C: LOW-DIMENSIONAL REPRESENTATION OF PROTEIN CONFORMATIONS

To perform a low-dimensional embedding of the conformational ensemble, strain is used as a measure of dissimilarity between two conformations. Specifically, we computed strain for 900 pairs of structures (30×30), corresponding to the five hexamers in the mechanochemical cycle ($s_1, \ldots, s_5$). The strain for each pair was calculated using the PSA parameters detailed in the Supplementary Methods.

After performing all pairwise comparisons, we constructed a pairwise strain matrix M, where each element represents the mean strain $\langle \epsilon_3 \rangle$ across all N atoms included in the analysis, $\langle \epsilon_3 \rangle = \sum_i^N \epsilon_{i,3}/N$. Since M is not necessarily symmetric, we symmetrised it as $\frac{1}{2}(M + M^{\mathrm{T}})$ to obtain a suitable dissimilarity matrix for multidimensional scaling (MDS) (see Fig 6 for a schematic overview). MDS then identifies a set of coordinates in a lower-dimensional space (here, two dimensions: (x, y)) for each row of the dissimilarity matrix.

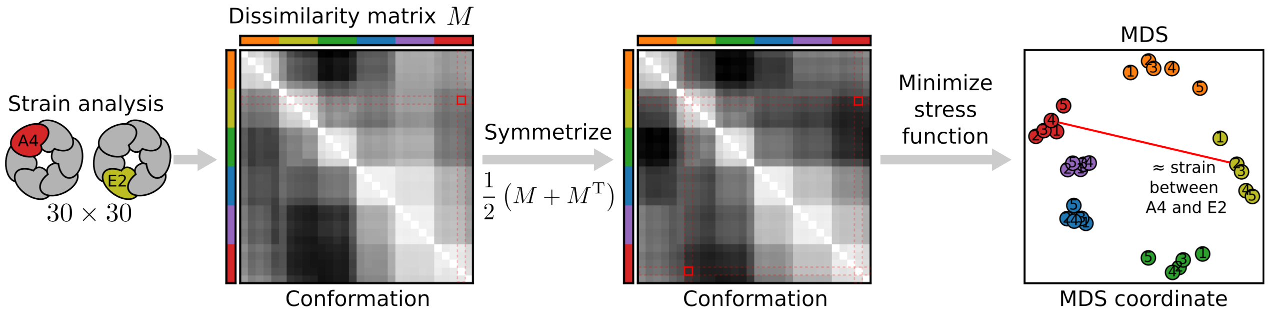

FIG. 6. *Workflow to build a low-dimensional embedding from structural comparisons.* The approach begins by calculating the local strain (or other measure of dissimilarity) for all possible pairs of conformations. In the figure, we highlight the structural comparison between conformations A4 and E2 as an example. We build a matrix M with rows and columns representing particular conformations, whose elements correspond to the mean strain over the whole structure. Darker shades of gray represent high mean strain. Because such matrix is not symmetric, we apply a symmetrisation operation. Finally, we compute the MDS for the symmetrised M as a dissimilarity matrix. This procedure is carried out by minimising a stress function, for which we considered an Euclidean distance on strain space.

Beyond the extensive strain ϵ_3, we explored additional dissimilarity metrics to assess their ability to order the mechanochemical cycle (Fig 7). These included the compressive strain ϵ_1, the total elastic pseudoenergy ΔE_{el}, and local rotation angles, all derived from PSA. The local rotation angles quantify the extent to which the local atomic neighborhood undergoes rigid body rotation, as illustrated by the comparison between the rotated ellipse and the sphere in Fig 1B. The intermediate eigenvalue ϵ_2 was not considered a suitable dissimilarity metric, as it includes both positive and negative strain values (reflecting expansions and contractions). For comparison, we also constructed low-dimensional representations using root-mean-square deviation (RMSD) with three different structural alignment strategies.

The alignment approaches used in these analyses are as follows: In Fig 7D, the entire RuvB assemblies were aligned; in Fig 7E, individual RuvB subunits were aligned; and in Fig 7C and F, the assemblies were aligned with an additional rigid body rotation in multiples of 60° about the central pore axis to approximately superimpose the conformations being compared.

Our results show that most metrics can capture the cyclic nature of RuvB conformations, but a clear and identifiable ordering is most apparent with ϵ_3 (Main text Fig 2C), ϵ_1 (Fig 7A), and RMSD (Fig 7F). Notably, both strain metrics are able to sort the cycle regardless of alignment strategy, whereas RMSD only provides a clear ordering under a specific and nontrivial alignment approach.

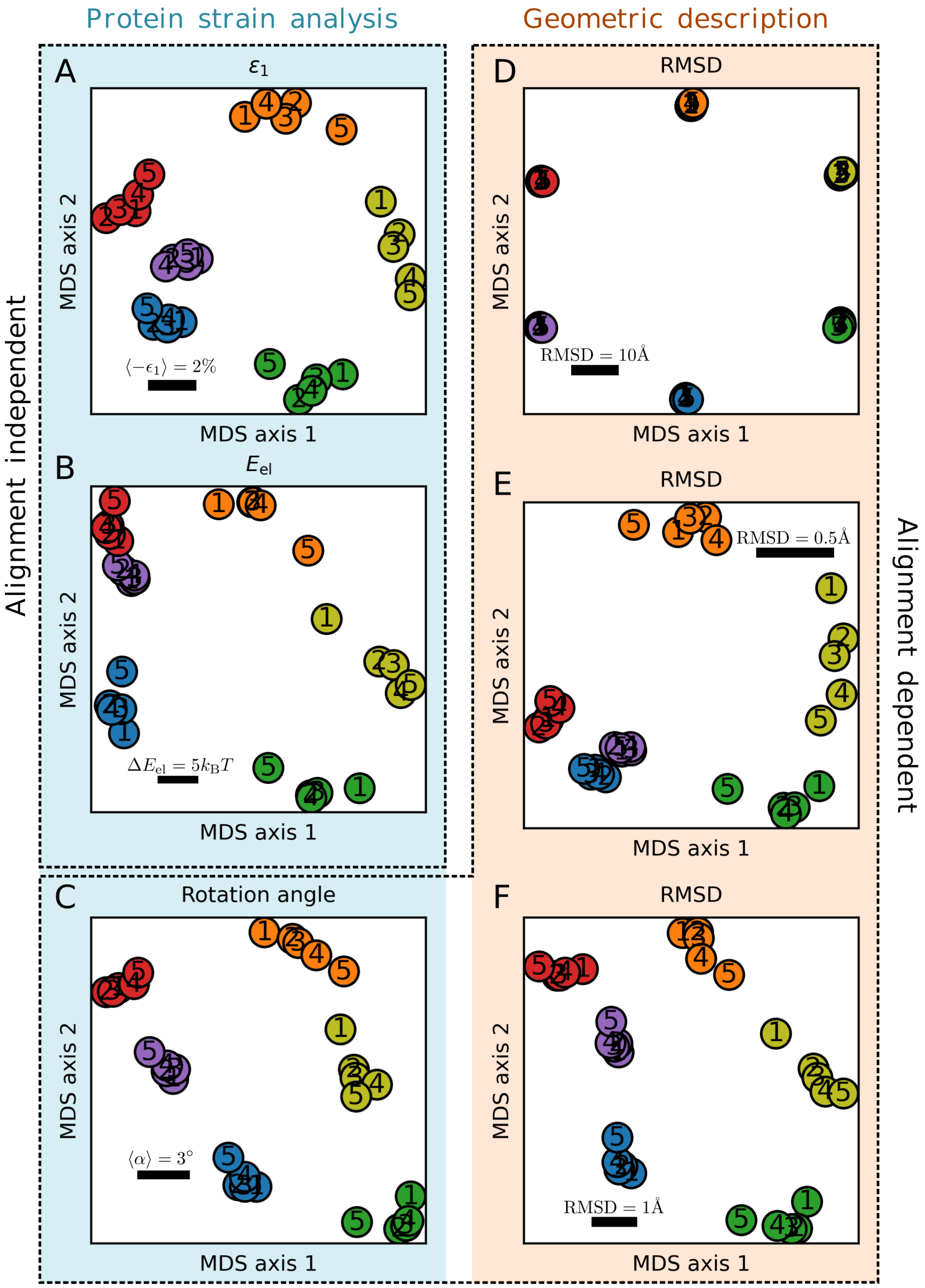

FIG. 7. *Multidimentsional scaling analyses performed with different metrics.* MDS analyses using **A.** The mean compressive strain (-ϵ_1) over the protein structure. **B.** The total elastic pseudoenergy ΔE_{el} of each comparison. **C.** The mean rotation angle α. The following panels display the root mean square deviation, RMSD, computed for three different structural alignment choices: **D.** aligning RuvB hexamers; **E.** aligning RuvB protomers; and **F** aligning RuvB hexamers and then performing rigid body rotations along the pore axis to superimpose conformations being compared.

APPENDIX D: ELASTIC PSEUDOENERGY ANALYSES WITH ALTERNATIVE REFERENCE STRUCTURES

The quantification of RuvB's elastic pseudoenergy landscape in this study relies on the choice of a reference structure to establish an energy baseline. This choice, we stress, must be biologically justified. For RuvB, we selected a reference structure (s_0) solved from the same cryo-EM dataset as the cycle conformations. Critically, s_0 exhibits two distinguishing features: (1) it binds ATP at the terminal position adjacent to DNA, and (2) it lacks interaction with RuvA, a key cofactor essential for efficient RuvB activity. These characteristics suggest s_0 represents a latent, pre-engaged state of the mechanochemical cycle [27]. This interpretation is further supported by the low-dimensional projection of conformations (Fig S1), which positions s_0 between s_5 and s_1.

To investigate how the choice of reference structure influences the results, we explored four additional RuvB hexameric structures as alternative references:

- 7PBQ: another potential initial state from the same EM dataset as s_0 but engaged with RuvA.
- 8EFV: A RuvB-DNA structure with 96% sequence coverage and 56% sequence identity to our study's constructs [28].
- AlphaFold 3 (apo): The top-ranked RuvB model (same sequence as our study) without DNA.
- AlphaFold 3 (DNA-bound): The top-ranked RuvB-DNA complex model (same sequence) [29].

The first two structures follow the same subunit labelling convention as the cycle conformations (A at the staircase top, interacting with DNA, followed by B, C, and D). In contrast, the AlphaFold models required reçlabeling to ensure consistency: the apo structure adopted a near-symmetric assembly, while the DNA-bound structure initially used divergent labels. After standardisation, we performed multidimensional scaling using extensive strain (ϵ_3) as the dissimilarity metric (Fig 8).

The results from the MDS analysis reveal distinct spatial relationships between the alternative reference structures and the cycle conformations. For example, the structure 7PBQ, much like s_0, clusters immediately before s_1 in the mechanochemical cycle, suggesting it could also serve as an initial state. In contrast, 8EFV is positioned further from the cycle conformations and does not cluster with any particular position, however, it tends to be closer to the cluster corresponding to each equivalent position than to others. The AlphaFold model generated in the absence of DNA forms a symmetric assembly, with all subunits adopting the same conformation; interestingly, this conformation resembles those found in position F (which is detached from DNA in the RuvB cycle). The DNA-bound AlphaFold model displays a more complex pattern, with some subunits matching their cognate positions (such as A and F), while the remaining subunits either form a separate cluster or do not cluster at all. Taken together, these observations indicate that only 7PBQ emerges as a plausible candidate for the initial state, closely mirroring the behaviour of s_0.

When constructing elastic pseudoenergy profiles using these alternative references, clear differences emerge in both the baseline and the shape of the resulting curves (Fig 9). For all references, there is a pronounced decrease in elastic pseudoenergy upon transition to position C, except in the case of the symmetric assembly. The overall height of the energy baseline reflects how closely related the reference and cycle conformations are. However, the behaviour of the curves for positions F, E, and D varies depending on the chosen reference. For instance, the 8EFV reference exhibits two large energy barriers between F1 and F5 and between D1 and D5, while the 7PBQ reference shows a gradual increase in elastic pseudoenergy from F1 to C4. These differences highlight how the choice of reference structure can influence both the quantitative and qualitative interpretation of the elastic pseudoenergy landscape.

To further assess whether the same regions of RuvB contribute to the total elastic pseudoenergy across different conformations, we identified regions of interest as described in the main text. Consistent with previous findings, the choice of reference structure influences which residues are highlighted, resulting in varying degrees of overlap with those identified using s_0 as the reference. Importantly, all four regions defined in the main text were represented when using alternative references, although the specific residues included within each region differed. Regions 3 and 4 were consistently identified across all reference structures, albeit with differences in the number and identity of residues. In contrast, Regions 1 and 2 were detected in all cases except when using 8EFV as the reference, which proved to be the most dissimilar among the structures examined.

The analysis using 7PBQ as the reference, which could have been a plausible alternative for the main text, revealed two notable distinctions. First, there were fewer variable residues identified in Region 3 compared to the original analysis. Second, a novel group of residues, not previously assigned to any region, was detected. This additional group, situated within the large ATPase domain, comprises residues that interact in *trans* with Region 4 throughout the cycle. These unique features likely contribute to the observed differences in the shape of the elastic pseudoenergy landscape when 7PBQ is used as the reference. Specifically, the cycle conformations exhibit pronounced deformation in Region 3, particularly at position E, while RuvA–RuvB interactions involving Region 4 are prominent at position

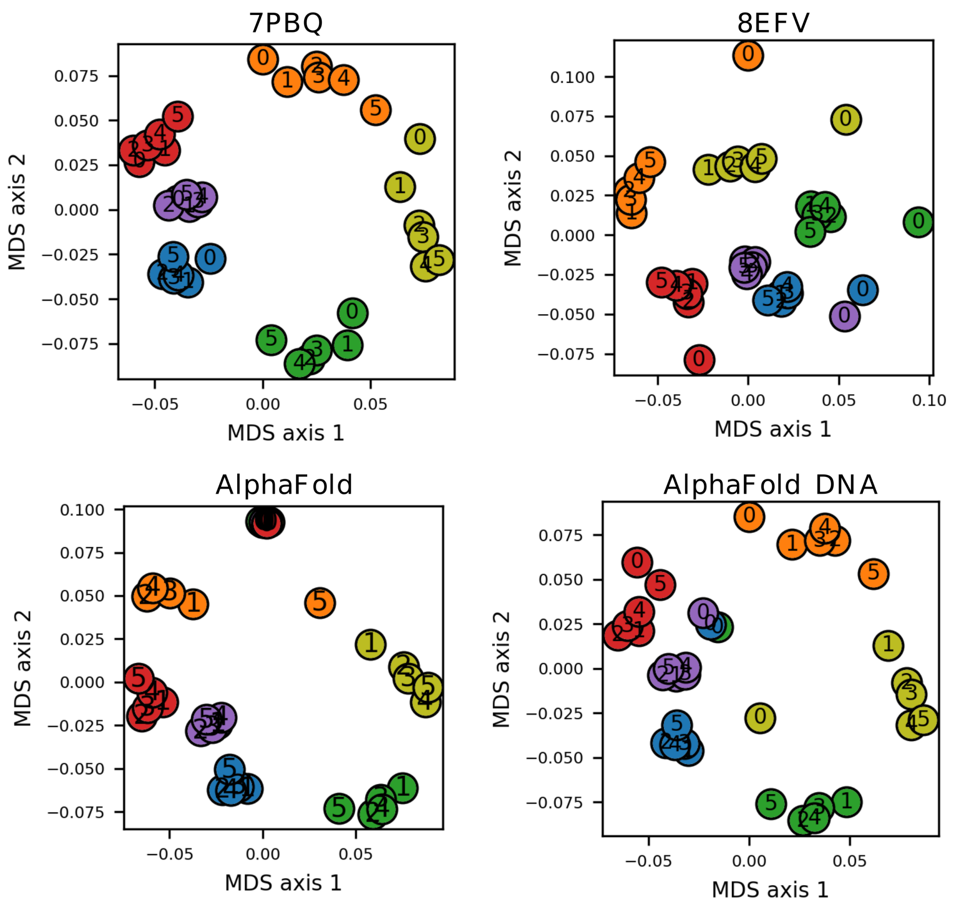

FIG. 8. *Multidimensional scaling analysis of pairwise strain with cycle and reference structures.* MDS plots using as dissimilarity metric $\langle \epsilon_3 \rangle$ between conformations. The reference conformations of each plot (shown in the title) are labelled as 0.

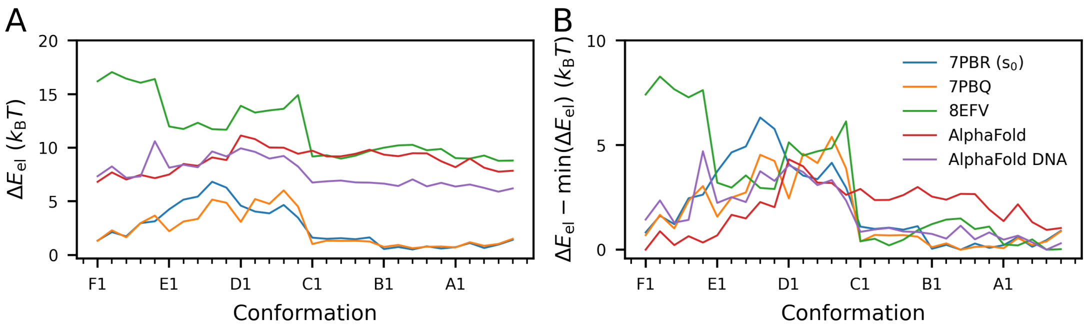

FIG. 9. *Pseudoenergy landscape of RuvB cycle using different references.* **A.** Total elastic pseudoenergy computed comparing cycle conformations to their cognate reference conformation. **B.** Amplitude of elastic pseudoenergy profile.

D. These structural distinctions align precisely with the regions of the pseudoenergy landscape where the profiles derived from 7PBQ and s_0 diverge.

Taken together, these results demonstrate that the choice of reference structure exerts a significant influence on the energetic characterisation of the RuvB conformational cycle. Nevertheless, the usage of our MDS-based approach provides a systematic means of evaluating and selecting biologically meaningful reference structures for further analyses.

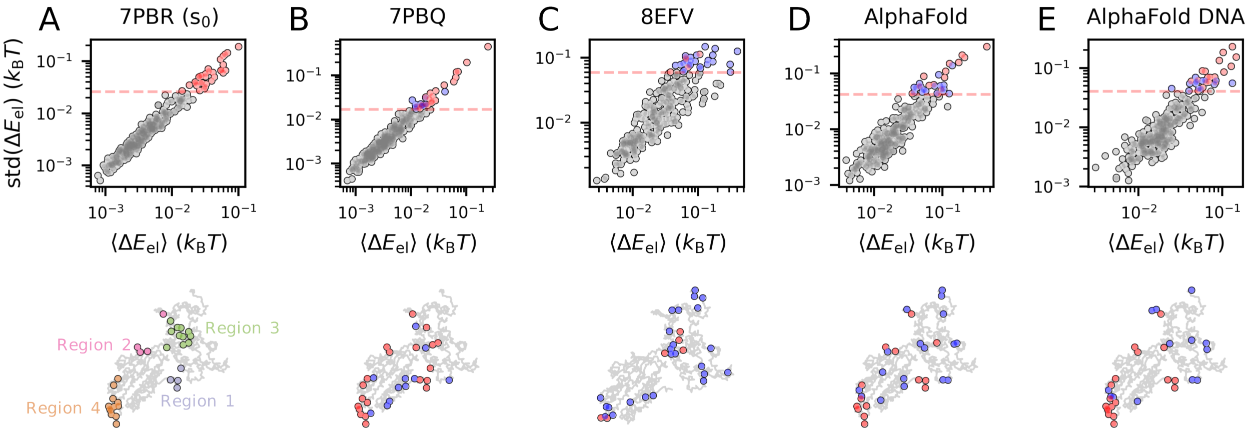

FIG. 10. *Identification of residues with most variable elastic pseudoenergy profiles using different references.* On top, we show the relationship between means and standard deviations of elastic pseudoenergy per residue. Below, we show the 10% most variable residues in a projection of a RuvB conformation. **A.** Mechanically active regions identified in the main text, using s_0 as reference. Residues are colored according with the scheme displayed in the main text. **B.** Most variable residues using 7PBQ as a reference shown in blue and red are compared with the mechanically active residues identified in the main text (first panel). Residues marked in red are common to the set identified for the reference s_0 and those in blue are not. In the following, the same procedure is repeated for references **C.** 8EFV, **D.** RuvB AlphaFold model, **E.** RuvB and DNA AlphaFold model.

APPENDIX E: EXPLORATION ENERGY-RELATED PARAMETERS IN THE KINETIC MODEL

In this study, we develop a kinetic model that connects the microscopic conformational cycle of RuvB to its macroscopic biophysical behaviour. The model integrates three energy contributions governing conformational transitions: an energy potential, defined by a state-dependent elastic pseudoenergy landscape derived from protein mechanics; intersubunit coupling, modelled as a Kuramoto-inspired synchronisation model between subunits; and a non-equilibrium driving force, representing the chemical potential of the ATP hydrolysis cycle. In this appendix, we study how the interplay between these three energetic parameters shapes the emergent translocation speed.

We begin by highlighting the key parameters that control the amplitude of each energetic term. To explore the role of potential energy amplitude, we define a generalised potential:

$$V = V_\alpha \frac{\Delta E_{\mathrm{el}}}{\max(\Delta E_{\mathrm{el}})} \quad ,$$

where V_α scales the amplitude of the potential energy landscape ΔE_{el} while maintaining its shape. The parameterisation used in the main text corresponds to $V_\alpha = \max(\Delta E_{\mathrm{el}}) \approx 7k_{\mathrm{B}}T$. The remaining parameters are defined as in the main text: K, representing the intersubunit coupling strength, and $\Delta\mu$, denoting the magnitude of the non-equilibrium drive. Figure 11 illustrates how these energetic parameters affect translocation speed.

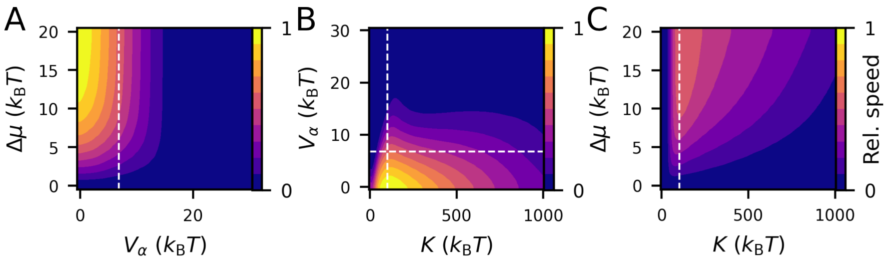

FIG. 11. *Dependence of relative processive speed on model parameters.* All heatmaps display the processive speed in colors normalised to the maximum speed achieved. **A.** Relative speed in function of potential energy amplitude and driving magnitude, at fixed coupling strength, adopted in the main text analyses ($K = 100k_{\mathrm{B}}T$). **B.** Relative speed in function of coupling strength and the potential energy amplitude, at fixed driving energy ($\Delta\mu = 20k_{\mathrm{B}}T$). **C.** Relative speed in function of coupling strength and driving magnitude, at fixed potential energy amplitude, matching main text results ($V_\alpha = \max(\Delta E_{\mathrm{el}})$). White dashed lines represent values used in the main text. All results show the mean speed of ten simulations, which were then smoothed with a Gaussian filter.

As shown in Fig 11A, the emergent speed of the system increases monotonically with $\Delta\mu$ and decreases with V_α. The driving energy biases the system towards an unidirectional progression, thereby increasing the motor's speed. For $K = 100k_{\mathrm{B}}T$, the speed saturates for $\Delta\mu > 15k_{\mathrm{B}}T$, as indicated by the vertical parallel level curves. Conversely, lower potential amplitudes reduce the energy barriers that individual particles must overcome to complete a full cycle, resulting in higher translocation speeds.

Figures 11B and C demonstrate that the translocation speed varies non-monotonically with the coupling strength, exhibiting a maximum around $100k_{\mathrm{B}}T$. At low coupling strengths, the trajectories of neighbouring subunits become uncoupled, preventing energy transduction between them. In the absence of coupling, particles rely solely on thermal fluctuations to cross the potential energy barrier. Conversely, at very high coupling strengths, translocation speed also declines. This decrease likely arises from a combination of finite-size effect (limited N) and the constraints imposed by Master-equation dynamics. As the coupling strength K increases, the system must overcome a larger collective energy barrier on E_{c} to perform a coordinated transition from state i to $i + 1$ across all subunits. Such an increased barrier inflates the time scale of successful forward transitions, thereby impairing overall processive speed.

APPENDIX F: CALIBRATION OF TIME SCALES WITH EXPERIMENTAL DATA

The kinetic model we introduced in the main text has the principle of detailed-balance as a key thermodynamic constraint. However, the actual form of the kinetic rates cannot be fully described by the system's energetics, as detailed-balance only dictates the behaviour of back-and-forth kinetic rates. We thus propose that a time scale variable controls the temporal dependency on kinetic rates, Eq. 8.

To calibrate such time scale, we use previously published data from two independent datasets that relate ATP concentration to translocation speeds [30]. In brief, Han and colleagues measured the [ATP]-speed curves with a bulk biochemical approach, hereby denoted as the biochemical data, and with a single-molecule experiment, resulting in what we denote biophysical data. We digitised both datasets using the web application `apps.automeris.io/wpd/`, reproduced in Fig 12A.

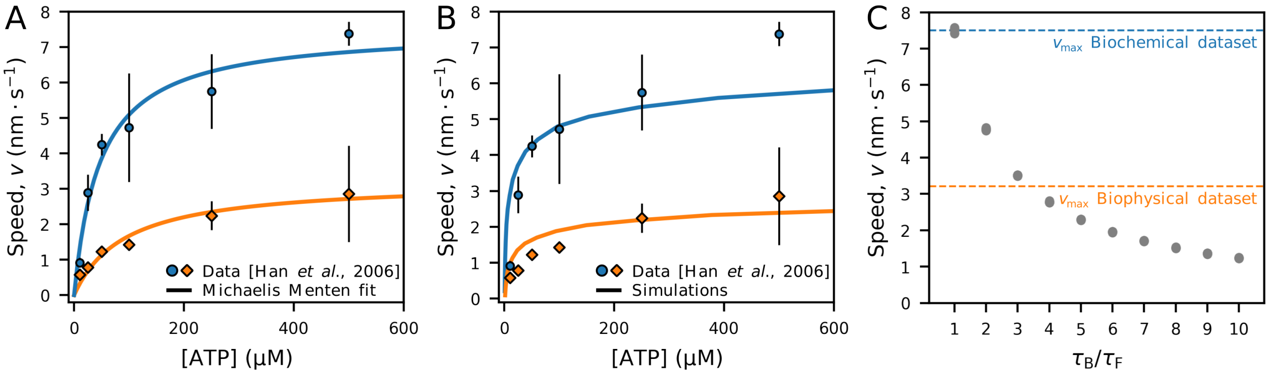

FIG. 12. *Fitting of characteristic time scale of conformational changes, τ.* **A.** Reproduction of data obtained experimentally in [30], where branch migration speed v is measured in two experimental approaches, namely *biochemical* and *biophysical*. **B.** Simulations adjusted to the experimental data. We sampled conformational trajectories with varying $\Delta\mu$ ranging from 0.2 to $9k_{\mathrm{B}}T$, each simulated with $n = 10$ different random seeds. **C.** Speed calculation for the biophysical dataset, with fixed τ_{F} and varying τ_{B} ($n = 10$ simulation replicates).

We define the time scales τ_{ab} in two forms to account for the specificities of each experimental approach. To model the biochemical dataset, we consider that all transitions have the same time scale, $\tau_{ab} = \tau_{\mathrm{F}}$. For the biophysical dataset, we model τ_{ab} as a step function to account for the slowed transitions due to the friction of the bead present in the experimental assay:

$$\tau_{ab} = \begin{cases} \tau_{\mathrm{F}} & \text{if } 0 < \theta_m, \theta'_m < \frac{2\pi}{3} \\ \tau_{\mathrm{B}} & \text{if } \frac{2\pi}{3} < \theta_m, \theta'_m < 2\pi \\ \frac{1}{2}(\tau_{\mathrm{F}} + \tau_{\mathrm{B}}) & \text{otherwise,} \end{cases}$$

with m representing the molecule which underwent a state transition in $\mathbf{a} \to \mathbf{b}$. θ_m and θ'_m are the coordinates of the molecule m before and after the transition, respectively. This particular choice of intervals in the reaction coordinate (0 and $\frac{2\pi}{3}$) for τ_{F} and τ_{B} corresponds to the RuvB conformations between free and bound. Therefore, this choice assumes that the viscous drag present in the experiment affects the transitions among DNA bound conformations. The transitions that display $\tau_{ab} = \frac{1}{2}(\tau_{\mathrm{F}} + \tau_{\mathrm{B}})$ are required to respect the symmetry condition $\tau_{ab} = \tau_{ba}$ for arbitrary neighbouring assembly states **a** and **b**.

We first calibrate τ_{F}, because it can be obtained directly from the biochemical dataset and our simulations. To do so, we extract the speed at saturated ATP concentration $v_{\max}$ fitting the biochemical dataset to the Michaelis-Menten curve $v = v_{\max}(1 + \frac{K_{\mathrm{M}}}{[\mathrm{ATP}]})^{-1}$, with v the translocation speed and K_{M} the Michaelis constant. We then adjusted τ_{F} to match the emergent translocation speed, simulating trajectories at saturated driving force ($\Delta\mu = 500k_{\mathrm{B}}T$, $n = 10$ simulation replicates), assuming that each cycle completion results in a translocation step of two DNA base-pairs, $d = 0.68$nm [27]. Next, we simulate multiple trajectories also at saturated chemical drive, with fixed τ_{F} and varying τ_{B}, in order to find τ_{B} that best reproduces $v_{\max}$ from the biophysical dataset, see Fig 12C. With this approach, we obtain the time scales estimates $\tau_{\mathrm{F}} = 1.13$ms and $\tau_{\mathrm{B}} \approx 3\tau_{\mathrm{F}} = 3.39$ms.

Finally, to adjust the speed-[ATP] curves, we derive from the definition of chemical potential $\Delta\mu = \log\big([\mathrm{ATP}]/C\big)$, where C depends on ATP, ADP, and $\mathrm{P_i}$ chemical potentials and concentrations. Then, we fit curves of processive speed simulated from a range of chemical potentials ($\Delta\mu \in [0.2, 9]k_{\mathrm{B}}T$) to the experimental data, optimising C and K_{M}, see

Fig 12B. Our fittings reveal a poorer match to the experimental data when compared to a Michaelis-Menten fitting, as the shape of the [ATP]-speed curve for the simulations presents a sharper transition to a saturated regime. Still, the curve we obtained recovers the trend obtained from the data for an interval of $\Delta\mu$ values within the expected range of driving chemical potential observed in bacteria. For instance, the free-energy difference from the ATP hydrolysis cycle has been estimated around $\Delta\mu_{\mathrm{Phos}} \approx 47\mathrm{kJ/mol} \approx 19 k_{\mathrm{B}}T$ for *E. coli* at exponential growth [31]. Such a value should represent an upper bound to the free-energy that can be used by a molecular motor relying on ATP hydrolysis for function. This is in line with the estimates we obtain, reaching saturation on translocating speed (numerically) at $15 k_{\mathrm{B}}T$.

APPENDIX G: SUPPLEMENTARY METHODS

PSA parameter choice for cycle reconstruction

We considered the following PSA parameter choice for all 900 structure pairs:

1. *Set of atoms:* heavy atoms of the main chain were selected, namely C_α, C, N, and O.

2. *Neighbourhood radius:* for a given conformation α, the set of atoms belonging to the local neighbourhood of i was defined as $\mathcal{N}_{\alpha,i} = \{j | \left\| \mathbf{\Delta x}_i^j \right\| < r\}$, with the cutoff radius $r = 9$Å;

3. *Neighbourhood method:* to enhance the local difference between conformations, we used a linear weight method among all conformations $w_j = n(j)/N$, where n is a function that counts the number of occurrences of $j \in \mathcal{N}_{\alpha,i} \forall \alpha \in \{\mathrm{F1}, \mathrm{F2}, \ldots, \mathrm{A5}\}$, in the $N = 30$ conformations. These weights represent the relative contribution of each atom in the neighbourhood to calculate the local deformation gradient.

PSA parameter choice for elastic pseudoenergy calculation

We considered the following PSA parameter choice to perform structural comparisons between cycle structures (s_1, s_2, s_3, s_4, s_5) and reference (s_0) as follows:

1. *Set of atoms:* heavy atoms of the main chain were selected, namely C_α, C, N, and O; the elastic pseudoenergy per residue consists of the sum of elastic pseudoenergy of its atoms.

2. *Neighbourhood radius:* for a given assembly α, the set of atoms belonging to the local neighbourhood of i was defined as $\mathcal{N}_{\alpha,i} = \{j | \left\| \mathbf{\Delta x}_i^j \right\| < r\}$, with the cutoff radius $r = 9$Å;

3. *Neighbourhood method:* to have a unified neighbourhood list per atom i, we considered the intersection of all neighbourhoods of i in each assembly, $\mathcal{N}_i = \mathcal{N}_{\mathrm{s}_0,i} \cap \mathcal{N}_{\mathrm{s}_1,i} \cap \cdots \cap \mathcal{N}_{\mathrm{s}_5,i}$. Thus, all neighbouring atoms in $\mathcal{N}_i$ have weight $w_j = 1$;

4. *Elastic constants:* for the material properties of proteins, we considered the Young modulus $Y = 100$MPa. See the Appendix B for further information.

5. *Protin volume:*. To estimate the total van der Waals volume V we used the software ProteinVolume [32] for each of the 36 RuvB conformations individually, Fig S2C.

Multiple sequence alignment and conservation

Multiple sequence alignment was used to map the amino acid sequence from this study to a set of 18 bacterial RuvB sequences listed in [33]. We use the software MEGA7 to perform the multiple sequence alignment, with the gap opening penalty set to 5 and the remaining settings set to default [34].

Model parameters and considerations

All simulations were conducted with $\delta = \frac{\pi}{3}$ and $K = 100 k_{\mathrm{B}} T$. We chose a value K that maximises translocation speed, see Appendix E. To compute the translocation speed, we considered that a translocation step of two base pairs (≈ 6.8Å) corresponded to a full cycle of a RuvB monomer. Also, the trajectories simulations were simulated with the initial condition $\boldsymbol{\theta}^\circ = \left[\theta_1(1), \theta_2(6), \theta_3(11), \theta_4(16), \theta_5(21), \theta_6(26)\right]$. We use this configuration because it represents an assembly state with conformations that correspond to s_1.

Definition of coupling energy and coupling force per state

To calculate the coupling energy as a function of conformation, we first need to introduce the probability density of the assembly states $P(\boldsymbol{\theta})$, with $\boldsymbol{\theta} = [\theta_1, \ldots, \theta_6]$. Because we are interested in describing the energetics of operational cycles, we consider here the probability density given that the initial condition $\boldsymbol{\theta}^{\circ}$ defined above. Therefore, we define $P^{\circ}(\boldsymbol{\theta}) = P(\boldsymbol{\theta}|\boldsymbol{\theta}(t=0) = \boldsymbol{\theta}^{\circ})$. Other particular choices of initial state can lead to local minima of the energy landscape which are not processive. The rationale for this choice is that RuvB assembly starts the mechanochemical cycle in s_1. With this, we introduce the procedure to calculate F, the mean value of an arbitrary function f evaluated in the reaction coordinates ϕ

$$F(\phi) = \langle f(\phi) \rangle_{\boldsymbol{\theta}} = \sum_{\boldsymbol{\theta}} P^{\circ}(\boldsymbol{\theta}) \left[\frac{1}{M} \sum_{m=1}^{M} f(\theta_m) \delta(\theta_m - \phi) \right] \quad ,$$

where δ is the Kronecker delta.

Using this definition, we then calculate the mean backward coupling $E_{\mathrm{c}}^{\mathrm{bwd}}$ as the mean elastic pseudoenergy $E_{\mathrm{c}}(\theta_m, \theta_{m+1})$ with the first argument fixed, and the mean forward coupling $E_{\mathrm{c}}^{\mathrm{fwd}}$ with the second argument fixed. Thus, we obtain

$$E_{\mathrm{c}}^{\mathrm{bwd}}(\phi) = \sum_{\boldsymbol{\theta}} P^{\circ}(\boldsymbol{\theta}) \left[\frac{1}{M} \sum_{m=1}^{M} E_{\mathrm{c}}(\theta_m, \theta_{m+1}) \delta(\theta_m - \phi) \right] \quad ,$$

$$E_{\mathrm{c}}^{\mathrm{fwd}}(\phi) = \sum_{\boldsymbol{\theta}} P^{\circ}(\boldsymbol{\theta}) \left[\frac{1}{M} \sum_{m=1}^{M} E_{\mathrm{c}}(\theta_{m-1}, \theta_m) \delta(\theta_m - \phi) \right] \quad .$$

The coupling forces are defined as the spatial derivative of the coupling energy. To compute the spatial derivative, we assume a linear relationship between conformational and physical spaces, $x_m = R\theta_m$, with the conformations arranged along a circumference of radius $R = 6$nm. Therefore, the coupling force can be computed as

$$F_{\mathrm{c}} = \frac{\partial}{\partial x} \left(E_{\mathrm{c}}^{\mathrm{bwd}} - E_{\mathrm{c}}^{\mathrm{fwd}} \right) = \frac{\partial \theta}{\partial x} \frac{\partial}{\partial \theta} \left(E_{\mathrm{c}}^{\mathrm{bwd}} - E_{\mathrm{c}}^{\mathrm{fwd}} \right) = R \frac{\partial}{\partial \theta} \left(E_{\mathrm{c}}^{\mathrm{bwd}} - E_{\mathrm{c}}^{\mathrm{fwd}} \right)$$

[1] G. N. Ramachandran, C. Ramakrishnan, and V. Sasisekharan, "Stereochemistry of polypeptide chain configurations," *Journal of Molecular Biology*, vol. 7, no. 1, pp. 95–99, 1963.

[2] Z. Sun, Q. Liu, G. Qu, Y. Feng, and M. T. Reetz, "Utility of B-Factors in Protein Science: Interpreting Rigidity, Flexibility, and Internal Motion and Engineering Thermostability," *Chemical Reviews*, vol. 119, pp. 1626–1665, feb 2019.

[3] S. A. Wankowicz, S. H. de Oliveira, D. W. Hogan, H. van den Bedem, and J. S. Fraser, "Ligand binding remodels protein side-chain conformational heterogeneity," *eLife*, vol. 11, p. e74114, mar 2022.

[4] Q. Dong, K. Wang, B. Liu, and X. Liu, "Characterization and Prediction of Protein Flexibility Based on Structural Alphabets," *BioMed Research International*, vol. 2016, no. 1, p. 4628025, 2016.

[5] C. Bohr, K. Hasselbalch, and A. Krogh, "Ueber einen in biologischer Beziehung wichtigen Einfluss, den die Kohlensäurespannung des Blutes auf dessen Sauerstoffbindung übt," *Skandinavisches Archiv Für Physiologie*, vol. 16, no. 2, pp. 402–412, 1904.

[6] W. A. Eaton, "A retrospective on statistical mechanical models for hemoglobin allostery," *The Journal of Chemical Physics*, vol. 157, no. 18, p. 184104, 2022.

[7] J. Monod, J.-P. Changeux, and F. Jacob, "Allosteric proteins and cellular control systems," *Journal of Molecular Biology*, vol. 6, no. 4, pp. 306–329, 1963.

[8] K. Imai, *Allosteric effects in haemoglobin.* Cambridge University Press, 1982.

[9] F. R. Smith and G. K. Ackers, "Experimental resolution of cooperative free energies for the ten ligation states of human hemoglobin.," *Proceedings of the National Academy of Sciences*, vol. 82, pp. 5347–5351, aug 1985.

[10] M. Perrella, A. Colosimo, L. Benazzi, M. Ripamonti, and L. Rossi-Bernardi, "What the intermediate compounds in ligand binding to hemoglobin tell about the mechanism of cooperativity," *Biophysical Chemistry*, vol. 37, no. 1, pp. 211–223, 1990.

[11] Y. Huang, M. L. Doyle, and G. K. Ackers, "The oxygen-binding intermediates of human hemoglobin: evaluation of their contributions to cooperativity using zinc-containing hybrids," *Biophysical Journal*, vol. 71, no. 4, pp. 2094–2105, 1996.

[12] T. Yonetani, S. Park, A. Tsuneshige, K. Imai, and K. Kanaori, "Global Allostery Model of Hemoglobin: MODULATION OF O₂ AFFINITY, COOPERATIVITY, AND BOHR EFFECT BY HETEROTROPIC ALLOSTERIC EFFECTORS *," *Journal of Biological Chemistry*, vol. 277, pp. 34508–34520, sep 2002.

[13] B. K. Biswal and M. Vijayan, "Structures of human oxy- and deoxyhaemoglobin at different levels of humidity: variability in the T state," *Acta Crystallographica Section D*, vol. 58, pp. 1155–1161, jul 2002.

[14] W. Bolton, J. M. Cox, and M. F. Perutz, "Structure and function of haemoglobin: IV. A three-dimensional Fourier synthesis of horse deoxyhaemoglobin at 5.5 Å resolution," *Journal of Molecular Biology*, vol. 33, no. 1, pp. 283–297, 1968.

[15] C. Tian, K. Kasavajhala, K. A. A. Belfon, L. Raguette, H. Huang, A. N. Migues, J. Bickel, Y. Wang, J. Pincay, Q. Wu, and C. Simmerling, "ff19SB: Amino-Acid-Specific Protein Backbone Parameters Trained against Quantum Mechanics Energy Surfaces in Solution," *Journal of Chemical Theory and Computation*, vol. 16, pp. 528–552, jan 2020.

[16] D. A. Case, H. M. Aktulga, K. Belfon, D. S. Cerutti, G. A. Cisneros, V. W. D. Cruzeiro, N. Forouzesh, T. J. Giese, A. W. Götz, H. Gohlke, S. Izadi, K. Kasavajhala, M. C. Kaymak, E. King, T. Kurtzman, T.-S. Lee, P. Li, J. Liu, T. Luchko, R. Luo, M. Manathunga, M. R. Machado, H. M. Nguyen, K. A. O'Hearn, A. V. Onufriev, F. Pan, S. Pantano, R. Qi, A. Rahnamoun, A. Risheh, S. Schott-Verdugo, A. Shajan, J. Swails, J. Wang, H. Wei, X. Wu, Y. Wu, S. Zhang, S. Zhao, Q. Zhu, T. E. I. I. I. Cheatham, D. R. Roe, A. Roitberg, C. Simmerling, D. M. York, M. C. Nagan, and K. M. J. Merz, "AmberTools," *Journal of Chemical Information and Modeling*, vol. 63, pp. 6183–6191, oct 2023.

[17] V. B. Chen, W. B. Arendall III, J. J. Headd, D. A. Keedy, R. M. Immormino, G. J. Kapral, L. W. Murray, J. S. Richardson, and D. C. Richardson, "*MolProbity*: all-atom structure validation for macromolecular crystallography," *Acta Crystallographica Section D*, vol. 66, pp. 12–21, jan 2010.

[18] M. R. Mitchell, T. Tlusty, and S. Leibler, "Strain analysis of protein structures and low dimensionality of mechanical allosteric couplings," *Proceedings of the National Academy of Sciences*, vol. 113, no. 40, pp. E5847—-E5855, 2016.

[19] E. Weinreb, J. M. McBride, M. Siek, J. Rougemont, R. Renault, Y. Peleg, T. Unger, S. Albeck, Y. Fridmann-Sirkis, S. Lushchekina, J. L. Sussman, B. A. Grzybowski, G. Zocchi, J.-P. Eckmann, E. Moses, and T. Tlusty, "Enzymes as viscoelastic catalytic machines," *Nature Physics*, 2025.

[20] M. Guthold, W. Liu, E. A. Sparks, L. M. Jawerth, L. Peng, M. Falvo, R. Superfine, R. R. Hantgan, and S. T. Lord, "A comparison of the mechanical and structural properties of fibrin fibers with other protein fibers," *Cell biochemistry and biophysics*, vol. 49, pp. 165–181, 2007.

[21] J. Howard, *Mechanics of Motor Proteins and the Cytoskeleton.* Sunderland, MA: Sinauer, 1 ed., 2001.

[22] A. P. Perrino and R. Garcia, "How soft is a single protein? The stress–strain curve of antibody pentamers with 5 pN and 50 pm resolutions," *Nanoscale*, vol. 8, no. 17, pp. 9151–9158, 2016.

[23] M. Dong, S. Husale, and O. Sahin, "Determination of protein structural flexibility by microsecond force spectroscopy," *Nature Nanotechnology*, vol. 4, no. 8, pp. 514–517, 2009.

[24] R. Afrin, M. T. Alam, and A. Ikai, "Pretransition and progressive softening of bovine carbonic anhydrase II as probed by single molecule atomic force microscopy," *Protein science*, vol. 14, no. 6, pp. 1447–1457, 2005.

[25] M. Radmacher, M. Fritz, J. P. Cleveland, D. A. Walters, and P. K. Hansma, "Imaging adhesion forces and elasticity of lysozyme adsorbed on mica with the atomic force microscope," *Langmuir*, vol. 10, no. 10, pp. 3809–3814, 1994.

[26] Y. Wang and G. Zocchi, "The folded protein as a viscoelastic solid," *Europhysics Letters*, vol. 96, no. 1, p. 18003, 2011.

[27] J. Wald, D. Fahrenkamp, N. Goessweiner-Mohr, W. Lugmayr, L. Ciccarelli, O. Vesper, and T. C. Marlovits, "Mechanism

of AAA+ ATPase-mediated RuvAB–Holliday junction branch migration," *Nature*, vol. 609, no. 7927, pp. 630–639, 2022.

[28] A. D. Rish, Z. Shen, Z. Chen, N. Zhang, Q. Zheng, and T.-M. Fu, "Molecular mechanisms of Holliday junction branch migration catalyzed by an asymmetric RuvB hexamer," *Nature Communications*, vol. 14, no. 1, p. 3549, 2023.

[29] J. Abramson, J. Adler, J. Dunger, R. Evans, T. Green, A. Pritzel, O. Ronneberger, L. Willmore, A. J. Ballard, J. Bambrick, S. W. Bodenstein, D. A. Evans, C.-C. Hung, M. O'Neill, D. Reiman, K. Tunyasuvunakool, Z. Wu, A. Žemgulytė, E. Arvaniti, C. Beattie, O. Bertolli, A. Bridgland, A. Cherepanov, M. Congreve, A. I. Cowen-Rivers, A. Cowie, M. Figurnov, F. B. Fuchs, H. Gladman, R. Jain, Y. A. Khan, C. M. R. Low, K. Perlin, A. Potapenko, P. Savy, S. Singh, A. Stecula, A. Thillaisundaram, C. Tong, S. Yakneen, E. D. Zhong, M. Zielinski, A. Žídek, V. Bapst, P. Kohli, M. Jaderberg, D. Hassabis, and J. M. Jumper, "Accurate structure prediction of biomolecular interactions with AlphaFold 3," *Nature*, vol. 630, no. 8016, pp. 493–500, 2024.

[30] Y.-W. Han, T. Tani, M. Hayashi, T. Hishida, H. Iwasaki, H. Shinagawa, and Y. Harada, "Direct observation of DNA rotation during branch migration of Holliday junction DNA by Escherichia coli RuvA–RuvB protein complex," *Proceedings of the National Academy of Sciences*, vol. 103, no. 31, pp. 11544–11548, 2006.

[31] Q. H. Tran and G. Unden, "Changes in the proton potential and the cellular energetics of Escherichia coli during growth by aerobic and anaerobic respiration or by fermentation," *European journal of biochemistry*, vol. 251, no. 1-2, pp. 538–543, 1998.

[32] C. R. Chen and G. I. Makhatadze, "ProteinVolume: calculating molecular van der Waals and void volumes in proteins," *BMC Bioinformatics*, vol. 16, no. 1, p. 101, 2015.

[33] H. Iwasaki, Y.-W. Han, T. Okamoto, T. Ohnishi, M. Yoshikawa, K. Yamada, H. Toh, H. Daiyasu, T. Ogura, and H. Shinagawa, "Mutational analysis of the functional motifs of RuvB, an AAA+ class helicase and motor protein for holliday junction branch migration," *Molecular microbiology*, vol. 36, no. 3, pp. 528–538, 2000.

[34] S. Kumar, G. Stecher, and K. Tamura, "MEGA7: Molecular Evolutionary Genetics Analysis Version 7.0 for Bigger Datasets.," *Molecular biology and evolution*, vol. 33, pp. 1870–1874, jul 2016.
