# Supplementary figures and images for: Elastic analysis bridges structure and dynamics of an AAA+ molecular motor
Source: PLoS Comput Biol. 2025 Oct 24;21(10):e1013596. doi: 10.1371/journal.pcbi.1013596 (PMC12582506; doi:10.1371/journal.pcbi.1013596)

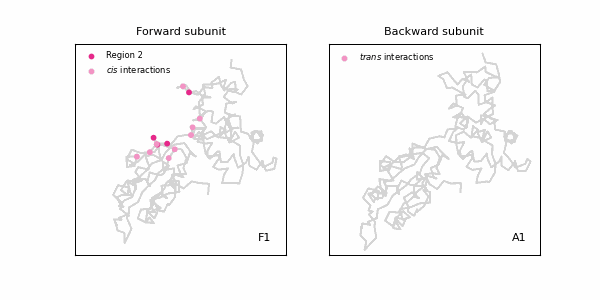

Supplement: S1 Scripts [file pcbi.1013596.s002.zip › movie/Region_2.gif]

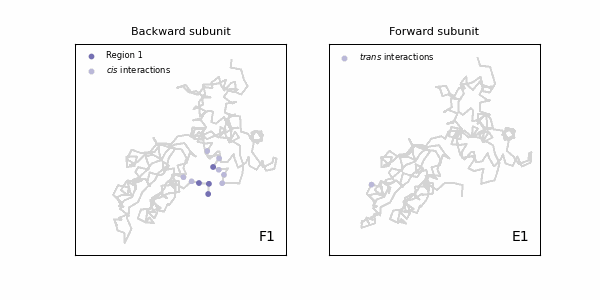

Supplement: S1 Scripts [file pcbi.1013596.s002.zip › movie/Region_1.gif]

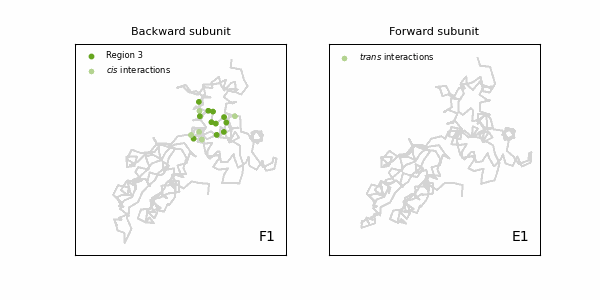

Supplement: S1 Scripts [file pcbi.1013596.s002.zip › movie/Region_3.gif]

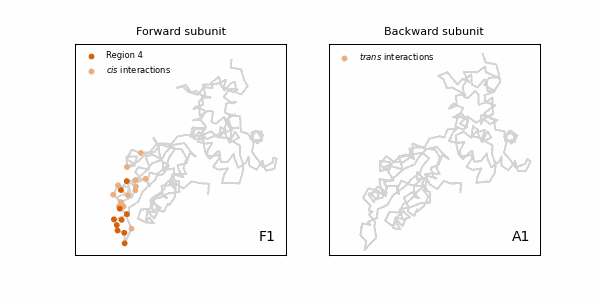

Supplement: S1 Scripts [file pcbi.1013596.s002.zip › movie/Region_4.gif]

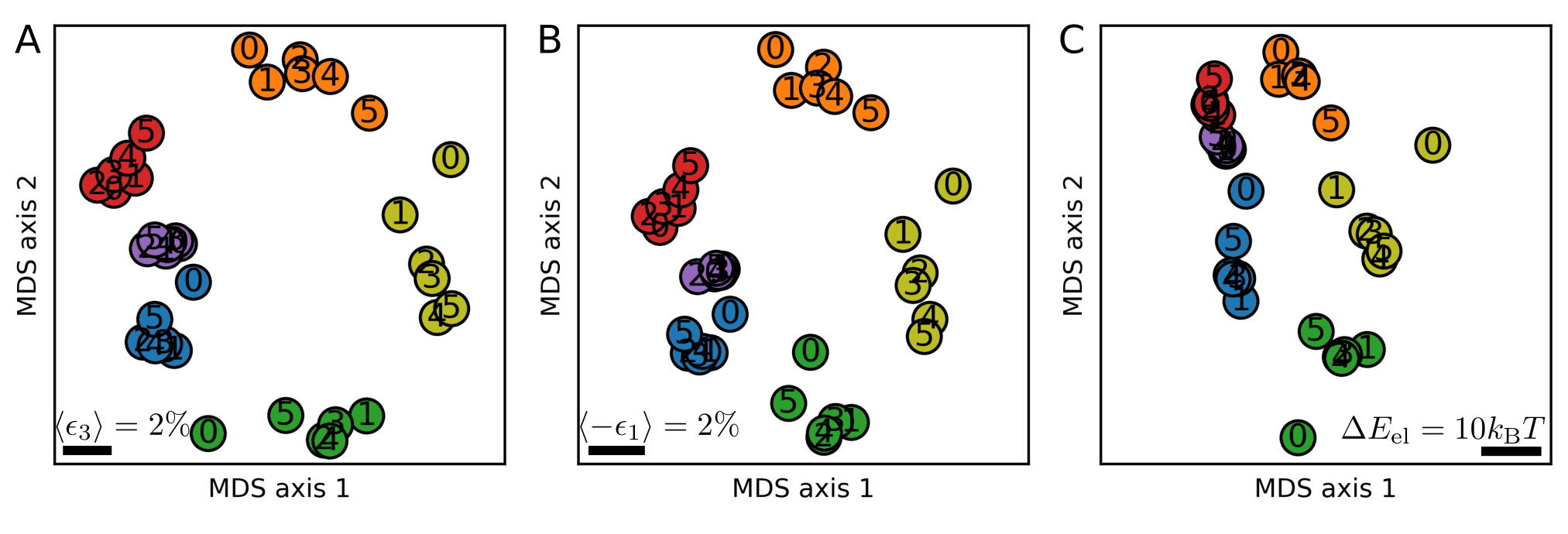

Supplement: S1 Fig — Multidimensional scaling analysis with cycle structures and the initial state s0 (mean of 100 MDS) using A. The mean largest eigenvalue of the strain tensor, ⟨ϵ3⟩, B. The mean lowest eigenvalue of the strain tensor, ⟨−ϵ1⟩, and C. The total elastic pseudoenergy ΔEel as dissimilarity measures. Despite most of the s0 conformations lying before s1 in clockwise order in this representation, the conformations in blue (corresponding to position C) and green (position D) are in different locations in MDS coordinates. That can be rationalised by the absence of interaction with RuvA in conformation C0 and the different nucleotide composition in position D (which harbours an ATP in conformation D0). The proximity of s0 and s1 for positions A, F and E suggests that s0 precedes s1 in order. However, the nucleotide composition of s0 subunits does not accommodate it as part of the mechanochemical cycle, suggesting that a priming ATP hydrolysis in D0 is required to start the mechanochemical cycle without a position switch. Note that in the cycle conformations, hydrolysis happens only in position A, as opposed to D. Therefore, considering s0 as an initial state not yet engaged in the mechanochemical cycle is a parsimonious interpretation of such MDS analyses. (TIFF) [file pcbi.1013596.s003.tiff]

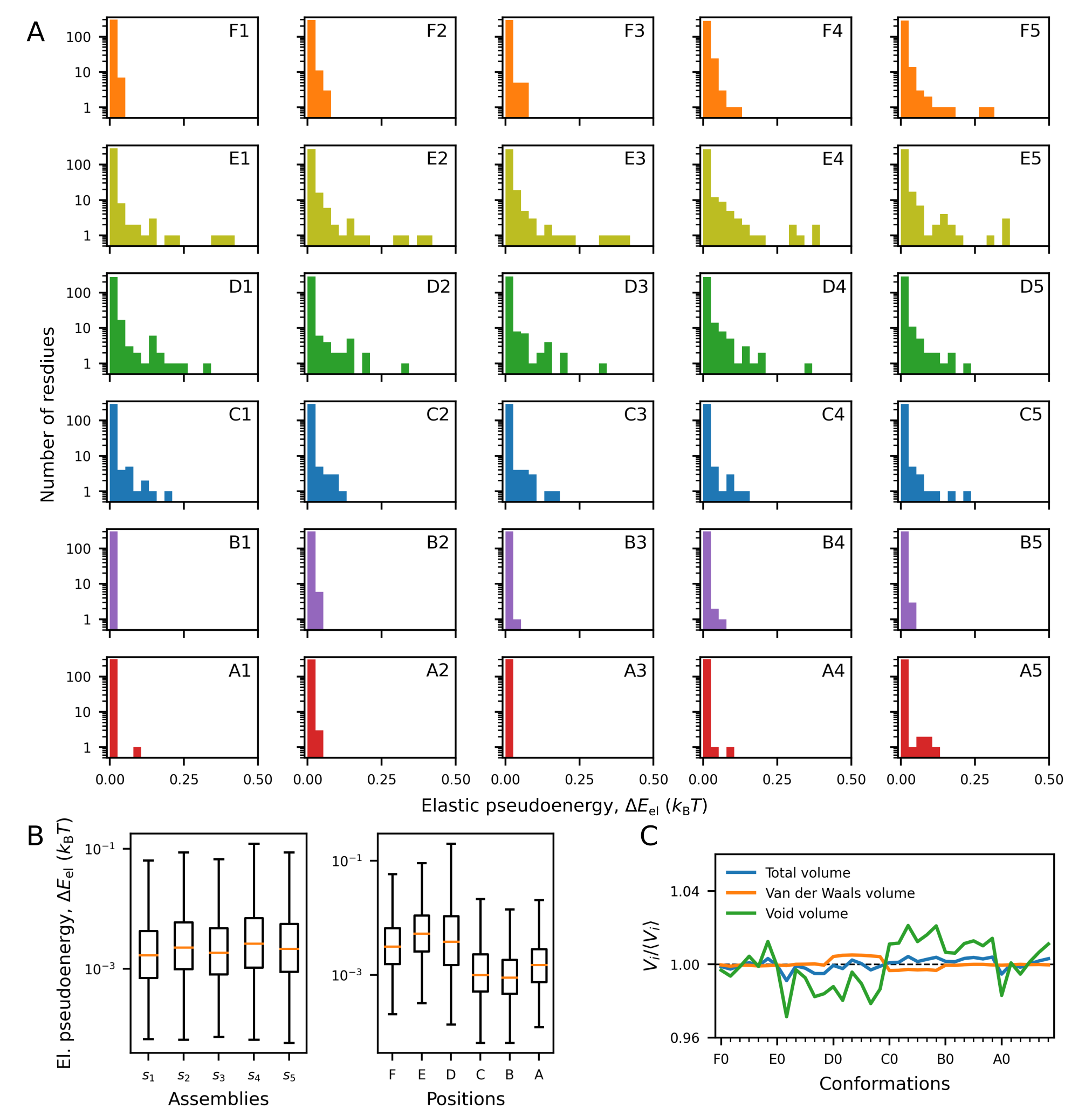

Supplement: S2 Fig — A. Histogram of elastic pseudoenergies per residues in all thirty monomeric conformations. The elastic pseudoenergy distribution, depicted in log scale, shows that ΔEel<0.01kBT for most residues in all conformations, with heavier tails for conformations in positions E and D. B. Total pseudoenergy distribution among assemblies is less variable than the pseudoenergy distribution among positions. C. Volume per conformation displays very low variation (around 0.1%) for the van der Waals volume, which was considered for the elastic pseudoenergy calculations. (TIFF) [file pcbi.1013596.s004.tiff]

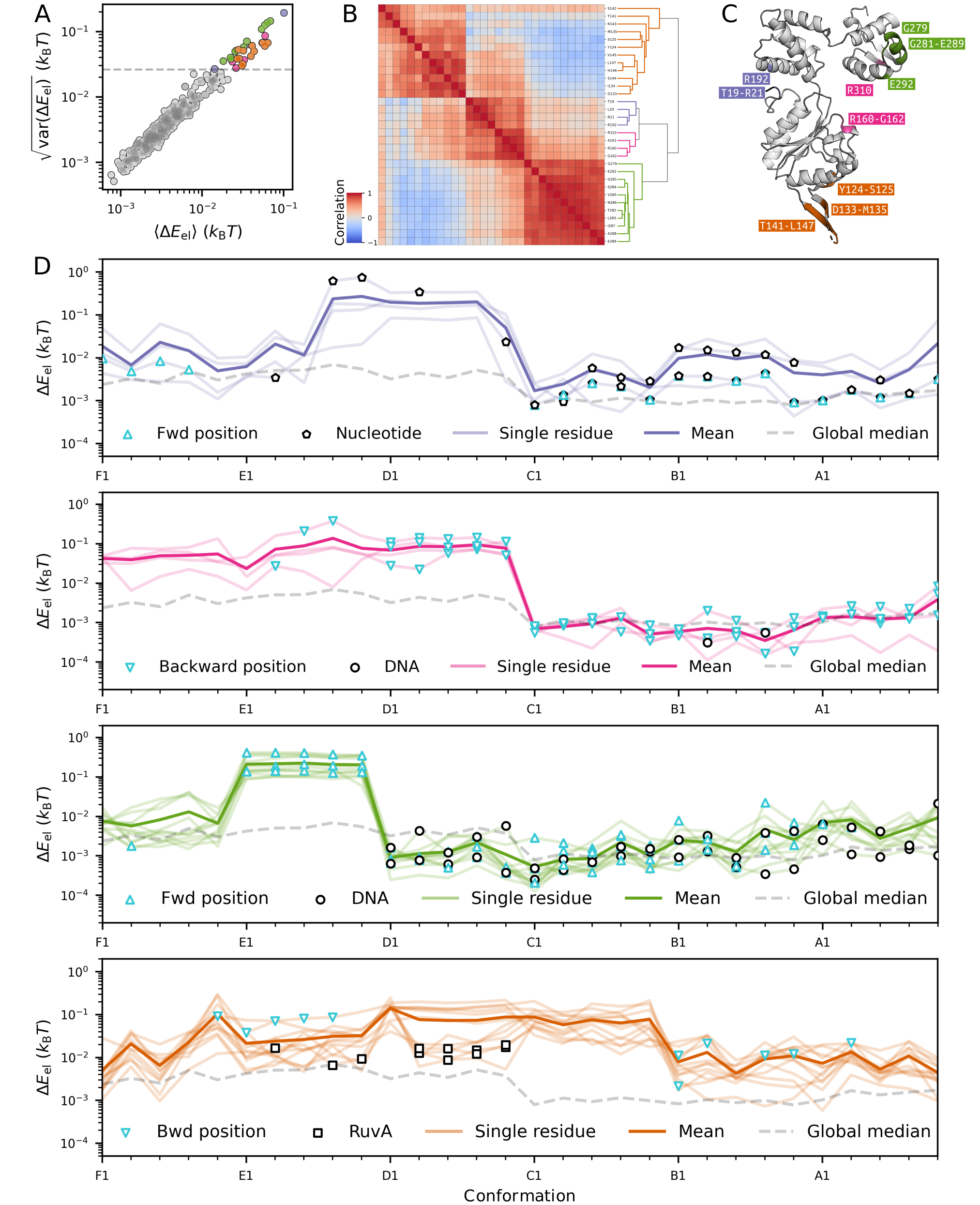

Supplement: S3 Fig — A. Mean and standard deviation of elastic pseudoenergy of residue trajectories are highly correlated. The dashed line defines a threshold on variance, selecting the top 10% more elastically variable residues. B. Correlation between elastic pseudoenergy profiles reveals clusters of residues with similar trajectories. The hierarchical clustering is based on the Euclidean distance among correlations. C. Mapping of spatial localisation of mechanically active regions. D. Elastic pseudoenergy profile of regions 1 to 4 and their specific intermolecular interactions. (TIFF) [file pcbi.1013596.s005.tiff]

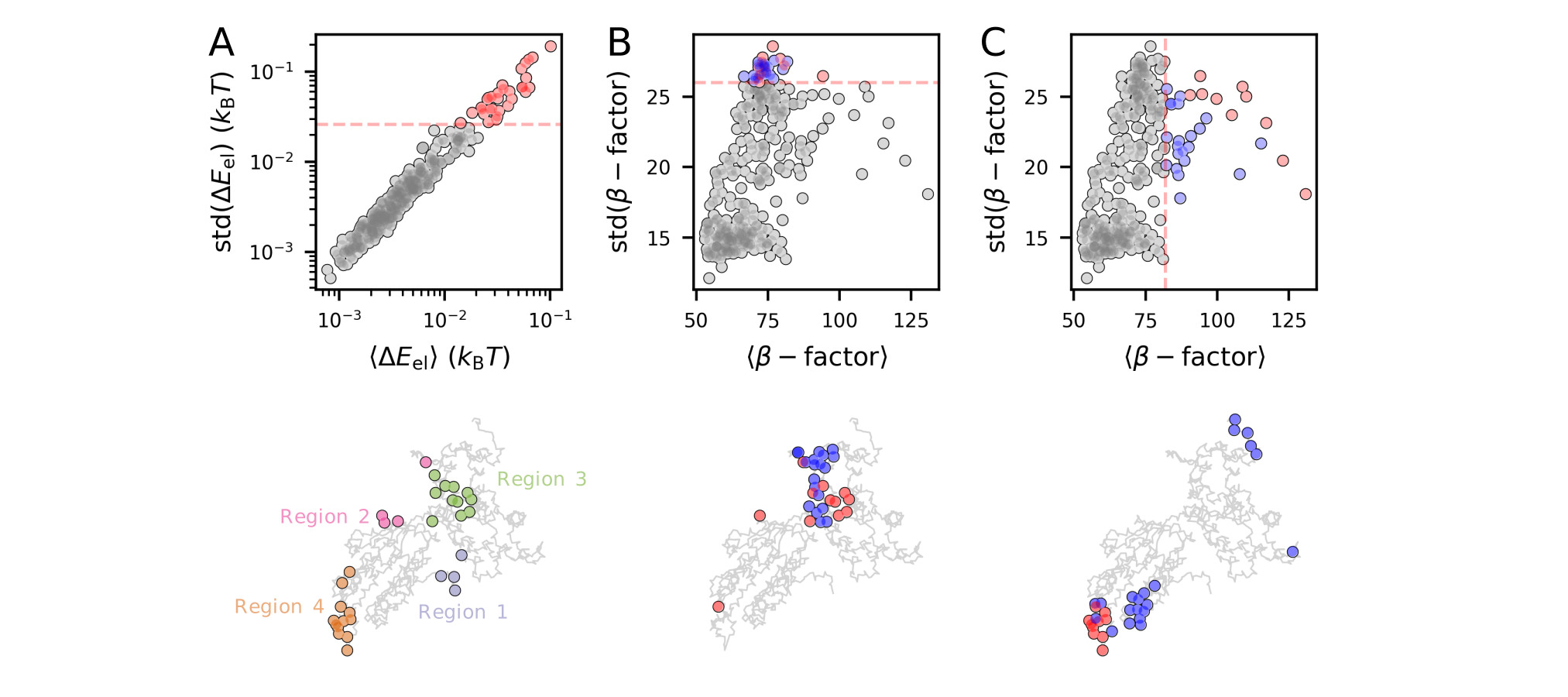

Supplement: S4 Fig — A. Approach used in the main text to identify regions of mechanical activity considering the top-10% residues on the standard deviation of elastic pseudoenergy. B. Regions of high β-factor standard deviation (top 10%). Residues marked in red agree with the elastic pseudoenergy criterion, whereas those in blue are unique to the β-factors standard deviation threshold. Residues identified above the threshold are highlighted in red and shown in a projection of RuvB monomer (below). Residues with high variability on β-factors are enriched with residues from Region 3 C. Regions of high β-factor means (top 10%). Residues with high mean β-factors are enriched with residues from Region 4. The results show that using β-factors does not fully recover the regions of interest identified through elastic pseudoenergy analysis, suggesting they should be interpreted as complementary rather than redundant metrics. (TIFF) [file pcbi.1013596.s006.tiff]

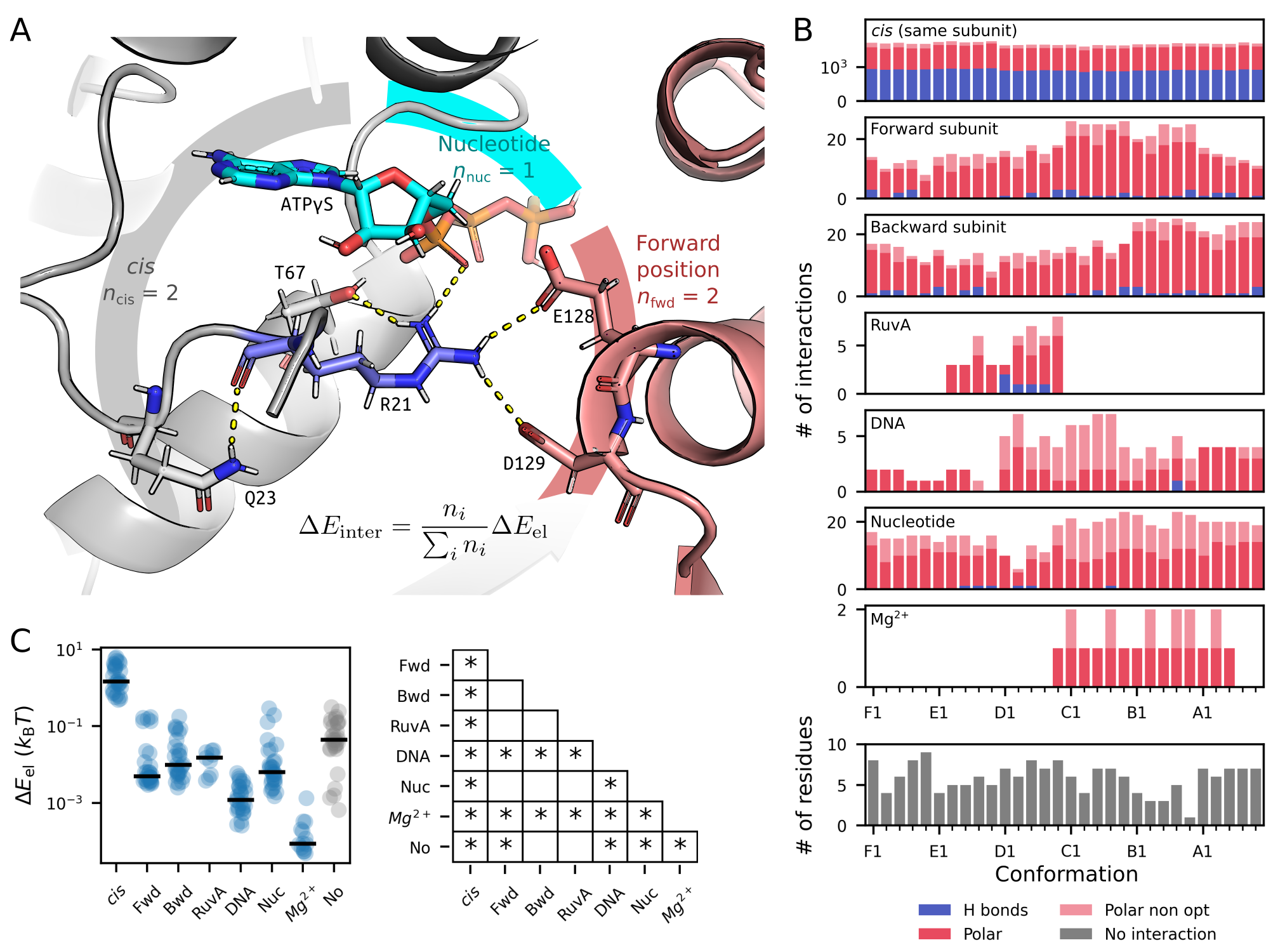

Supplement: S5 Fig — A. Example of polar interactions identified for R21 (in mechanically active region 1), which displays interactions in cis, with the forward RuvB subunit, and with the nucleotide. The elastic pseudoenergy of interaction ΔEinter is calculated as the average of the elastic pseudoenergy of the residue weighted by the number ni of interactions performed. B. Number of polar interactions per state grouped by type of interaction. Few residues do not display any of the considered types of polar interactions. C. Total elastic pseudoenergy with intra and intermolecular interactions and its associated statistics. We considered only the states where there was at least one interaction. On the right, we show the statistics tested between pairs of groups (Mann-Whitney U test, * corresponds to p < 0.05, Bonferroni corrected). (TIFF) [file pcbi.1013596.s007.tiff]

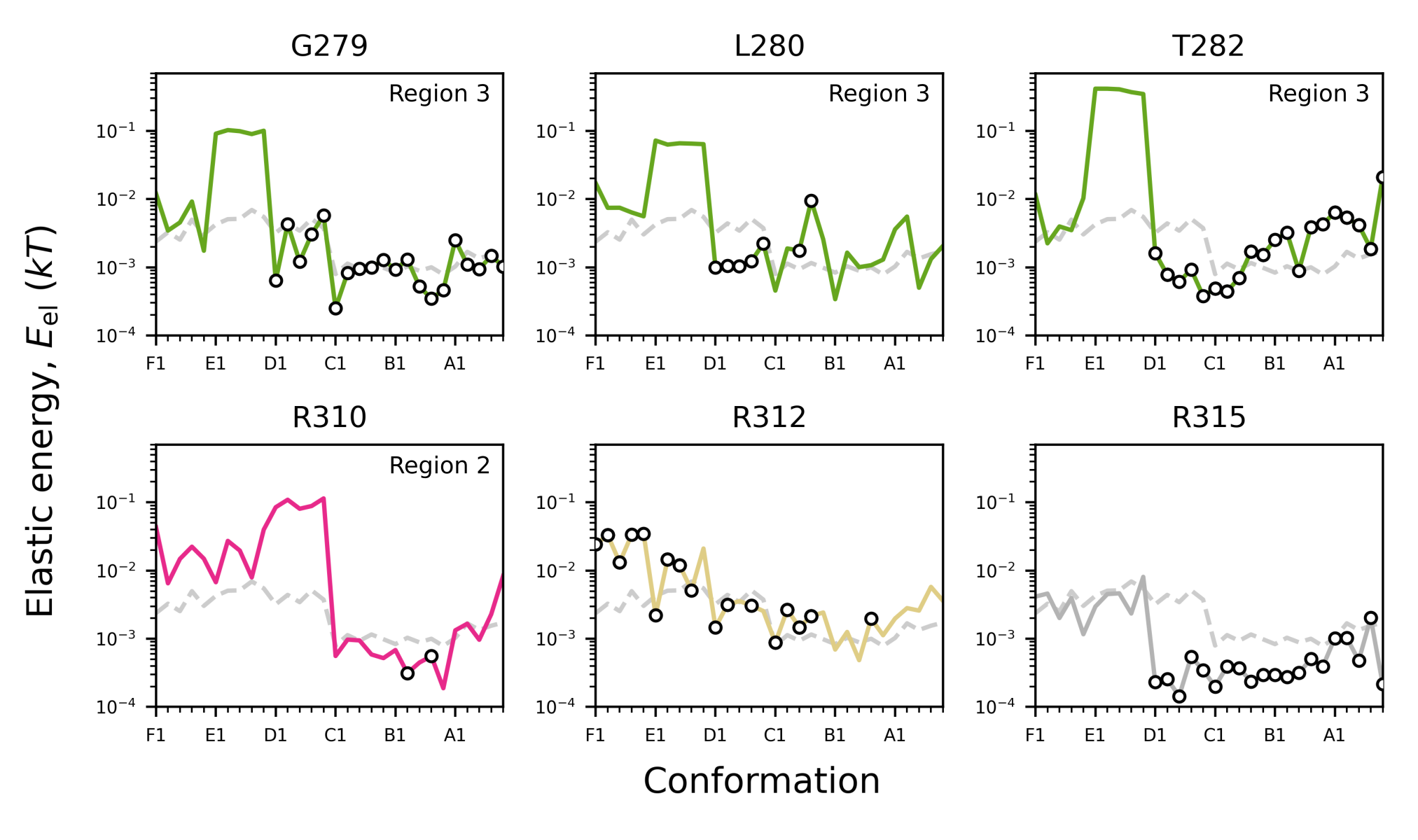

Supplement: S6 Fig — Elastic pseudoenergy quantification for residues that interact with the DNA via polar interactions in at least one conformation. Circle markers indicate states with predicted interactions, and the dashed line represents RuvB’s median elastic pseudoenergy profile. (TIFF) [file pcbi.1013596.s008.tiff]

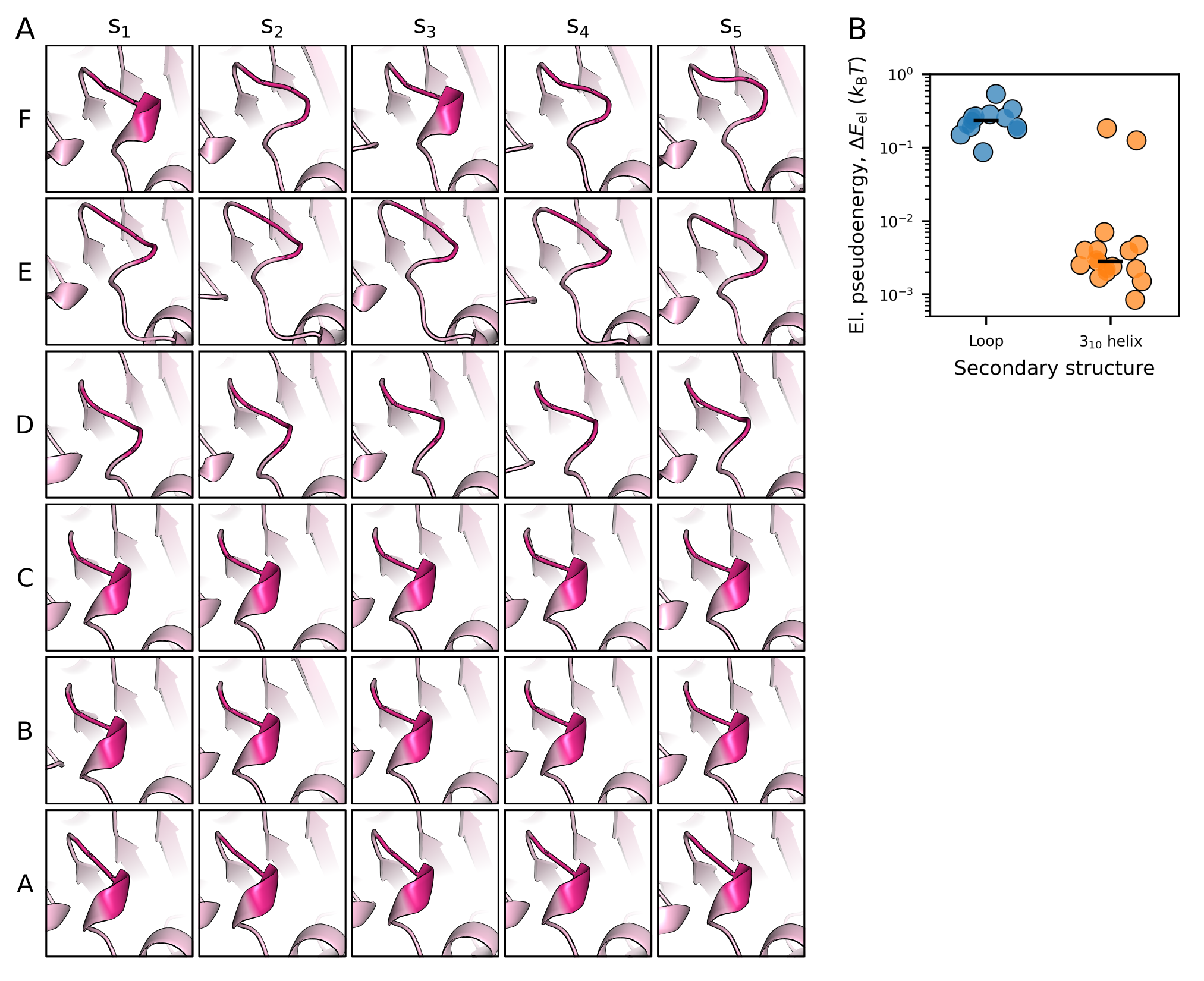

Supplement: S7 Fig — A. The residues highlighted in magenta represent part of the mechanically active region 2, found by our approach. Conformations from F to D, except for F1 and F3, were predicted to have a loop as a secondary structure, whereas conformations from C to A display a transient 310 helix. B. The total pseudoenergy of residues R160, A161, and G162 is shown according with the secondary structured. The total pseudoenergy of these residues when found on loop conformation is significantly different than when on 310 helix conformation (Mann-Whitney U test, p < 0.05). (TIFF) [file pcbi.1013596.s009.tiff]

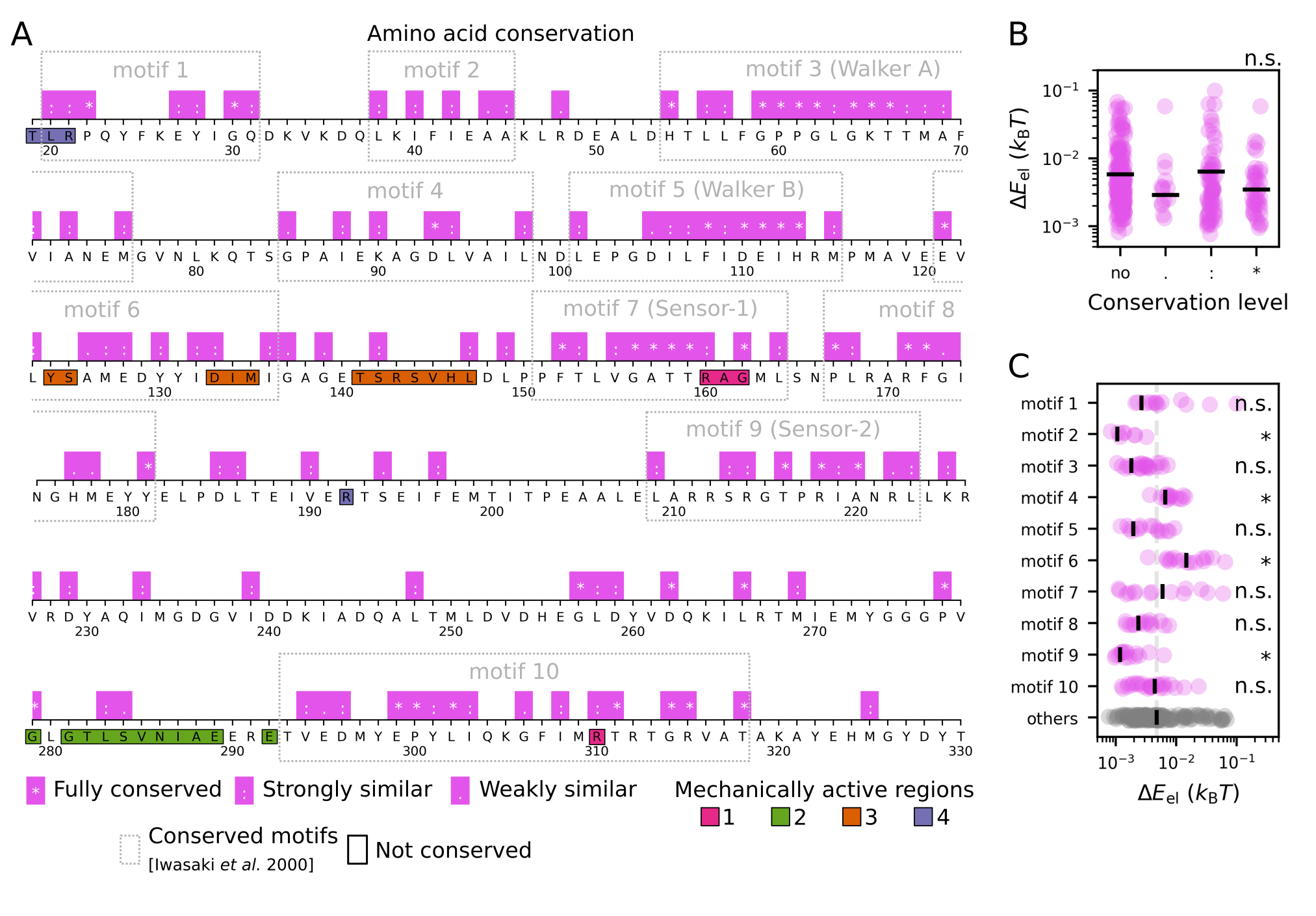

Supplement: S8 Fig — A. Map of amino acid conservation among RuvBs from 18 bacterial species [90] and the construct we analysed. The conserved motifs correspond to regions previously assigned by [90], to which the extent of conservation ranges from RuvB-specific to P-loop NTPases. B. Sequence conservation is not correlated with the mean elastic pseudoenergy per residue (Kruskal-Wallis H-test, p > 0.05). C. Mean elastic pseudoenergy of residues belonging to different conserved motifs. We found that the groups are significantly different (Kruskal-Wallis H-test, p < 0.05), but the pattern is not consistent across the different motifs. Motifs 2 and 9 were less energetic than non-conserved residues, whereas Motifs 4 and 6 were more energetic. Note that Walkers A and B (motifs 3 and 5) are not significantly more or less strained than non-conserved regions. (Mann-Whitney U-test for each motif vs other residues, Bonferroni corrected p < 0.05). (TIFF) [file pcbi.1013596.s010.tiff]

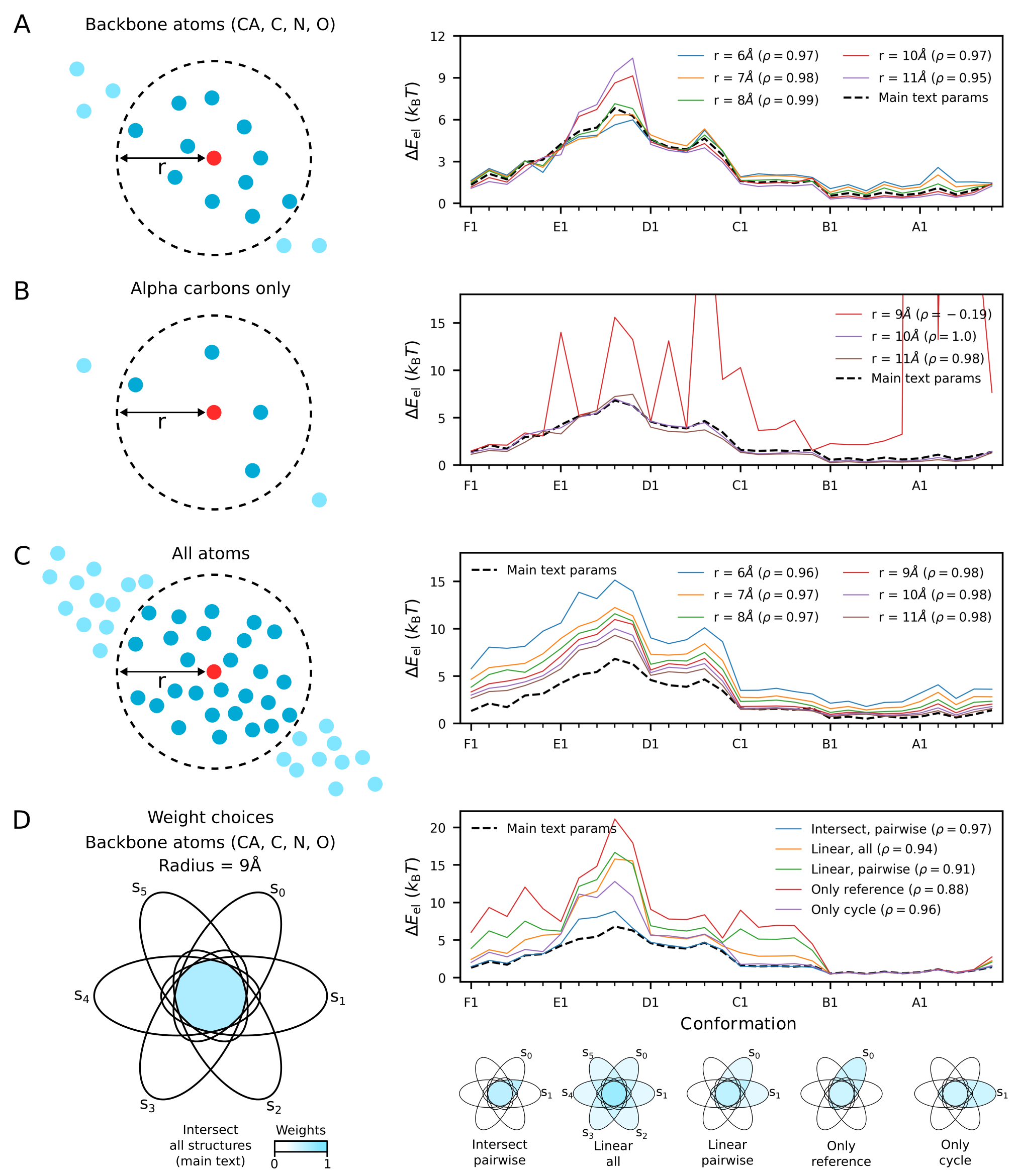

Supplement: S9 Fig — We systematically repeated the calculations for elastic pseudoenergy at different states, changing the atoms selected for the analysis, the radius of the local neighbourhood, and the method to assign weights to the atoms in the neighbourhood. We show the Pearson’s correlation (ρ) between the pseudoenergy landscapes obtained with different parameter choices and the main text analysis (backbone atoms, r = 9Å, intersect all structures). Overall, the main text parameter choice agrees qualitatively and quantitatively with other choices being, in general, more conservative (typically is as deformed as or less deformed than other pseudoenergy landscapes). All profiles display a similar trend, except for alpha carbons and radius r = 9Å due to imprecise calculations when the number of atoms in the local neighbourhood is small. A/B/C. elastic pseudoenergy profiles change the radius of the neighbourhood and the atom selection criteria. For these tests, we use the same weighting method from the main text. D. elastic pseudoenergy profile for different weight method, using backbone atoms and radius to 9Å. The Venn diagrams represent the atoms in the neighbourhood of radius r for different structures si, for the example s1 vs s0. (TIFF) [file pcbi.1013596.s011.tiff]

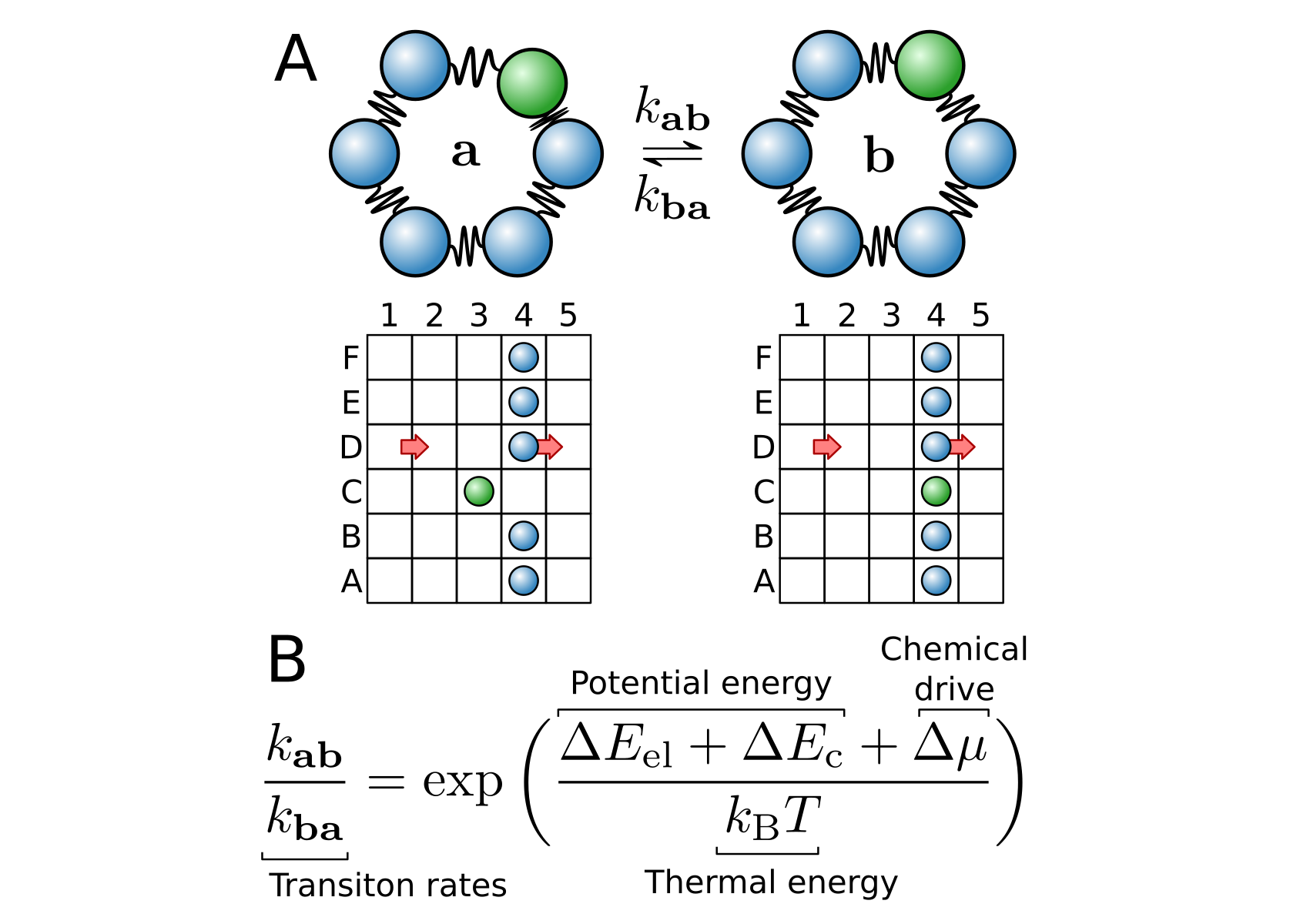

Supplement: S10 Fig — A. Representation of a particular transition between assembly states. A RuvB assembly consists of six coupled subunits, represented by circles connected through springs. In a given hexameric state, each subunit occupies one of the 30 conformations of the mechanochemical cycle, see tables below schematics for the example states a and b. Transitions among adjacent states are controlled by the kinetic rates k. In the example, the green circle transitions between the adjacent conformations C3 and C4, where the other subunits remain in the same configuration. Red arrows highlight the transitions driven by the chemical potential Δμ, which are associated with ADP release and ATP binding B. The detailed-balance condition enforces the ratio of back-and-forth transition rates to respect the energy difference of the assembly configurations. The driven transitions, however, are biased towards one direction. (TIFF) [file pcbi.1013596.s012.tiff]
