# Supplementary material for: Elastic analysis bridges structure and dynamics of an AAA+ molecular motor
Source: PLoS Comput Biol. 2025 Oct 24;21(10):e1013596. doi: 10.1371/journal.pcbi.1013596 (PMC12582506; doi:10.1371/journal.pcbi.1013596)
Supplement: Attachment — Submitted filename: Response_to_Reviewers.pdf [file pcbi.1013596.s018.pdf]

Response to referees

Letter to editors

Dear Alexander MacKerell and Arne Elofsson,

Thank you for considering our manuscript for publication in PLOS Computational Biology.

We are grateful for the referees' careful evaluation and their appreciation of our work. Both referees recommended publication. Referee #1 has made two specific suggestions to improve the manuscript. We have addressed these in detail below and revised the manuscript accordingly, which we believe is now clearer. We hope that the revised version is now suitable for publication in PLOS Computational Biology.

All the best,
Victor Mello, Jiri Wald, Thomas Marlovits, and Pablo Sartori.

Referee #1

In this manuscript, the authors present a detailed application of the innovative concept of "pseudoenergies" that they introduced previously. As their protein of choice, they focused on the conformational changes of RuvB during its hand-over-hand cycle along DNA. The paper has two parts: one is a sophisticated data analysis of the structural changes and associated energies, and the other is a kinetic non-equilibrium model of its function. The model partly incorporates insights from the first part, but makes non-trivial predictions that are cross-validated. The paper's conclusions are substantially supported by the supplementary material, and the limitations are clearly discussed.

This study is an original and substantial contribution to the open question of how to connect protein structure to function. Therefore, I recommend publication in PLOS CB

I have a few recommendations for the authors:

It is essential to note that it is fundamentally impossible to obtain kinetic information from static measurements, such as structures. Therefore, I was first very confused when I read the sentence in line 138. For me, it somehow implies that the cycle can be determined from ordering the conformations according to a principle of minimal elastic deformation. For a non-equilibrium system that cycles through a pathway of discrete states, the energy barriers (not the energy differences) between the states dictate the dynamics. It would be helpful if the authors could clarify that the elastic pseudoenergies can only contribute to equilibrium considerations, and insights into non-equilibrium phenomena must come from dynamic measurements. Therefore, it is surprising to me that the minimal elastic deformation principle suggests a reasonable sequence of states. It could also be a general principle in biology that I am not aware of. It would be interesting to discuss if there is a case of a sequence of conformational states that cannot be revealed from the minimum pseudoenergy principle. Perhaps it is possible to construct a simple example in which the energy barriers are chosen such that the cycle does not follow the minimum difference in internal energies between the states.

Response:
We thank the referee for raising the concern about the possible consequences of a protein operating far from equilibrium on applying the principle of minimum deformation. While we acknowledged that kinetics cannot be obtained from static snapshots, as stated in line 393, we indeed never explicitly relate this to the conformation ordering algorithm. We also recognise that our original phrasing may have suggested that the minimal deformation principle necessarily yields the correct time order, which was not our intention.

In our understanding, the "principle of minimum deformation" relies on the assumption that conformational states are sampled from a continuous cyclic trajectory, such that states close along the reaction coordinate are structurally more similar than distant ones. Thus, if a structural dataset provides sufficient and well-distributed snapshots of a given cycle, it

becomes possible to reconstruct its order. This was possible to achieve with our dataset because of the large number of conformations and the sequential nature of RuvB's subunit cycle.

As the referee observes correctly, non-equilibrium conformational transitions often display large deformations, such as fast powerstrokes. In cases for which this corresponds to a large component of the conformational cycle, where no intermediate steps can be recovered experimentally, it is unlikely that a strain-based approach can establish the order relationship between conformations.

Changes:
We have modified the text in the "Cycle reconstruction using minimal deformation principle" results section and in the discussion to clarify that such a method is not guaranteed to recover the time-ordered conformational cycle.

> *It is worth noting that the factor 2 in Eq. (7) is a choice, because detailed balance also holds for other ways of distributing "the driving" either to the forward or the backward rate.*

Response:
We appreciate the referee's request to make such a choice clear for the reader. Indeed, not only the driving force but also the definition of kinetic rate in all the three cases is subject to different choices that satisfy the local detailed-balance condition.

Changes:
We included a sentence in the Methods section "Stochastic kinetic model of RuvB assembly" to clarify that k_{ab} was a choice.

We thank the referee for the appreciation of our work and for the very thoughtful suggestions. We believe that these changes contributed significantly to the clarity of our text and to convey possible caveats to our reader that intends to adopt these approaches to their own datasets. We hope that these changes answer the referee's concerns.

Referee #2

RuvB is an ATPase protein that acts as a motor to drive branch migration of the Holliday junction in bacterial homologous recombination. While branch migration of the Holliday junction is taught in all molecular biology courses, the biophysical basis and exact mechanistic aspects have not been fully worked out. The convergence of the growing collection of well resolved single particle cryoEM structures, and advances in algorithms and GPU computing nodes make this erstwhile problem a good target.

The manuscript, “Elastic analysis bridges structure and dynamics of an AAA+ molecular motor,” by VH Mello et al. offers a plausible and accessible model. The authors relate stepwise structural transitions with energy obtained from chemical energy through hydrolysis of ATP to ADP and inorganic phosphate. the hydrolysis of ATP (adenosine triphosphate) into ADP (adenosine diphosphate) and an inorganic phosphate, Pi. Structural changes are expressed as elastic deformations quantified as mechanical energy using Hooke’s Law. The energy released is used to push the substrate (DNA) through the protein, RuvB, the helicase. The treatment is sound and consistent.

The model is laid out in the early figures (Figs. 1 and 2), and the results follow in Figure 3 displayed as a per residue plot of a subunit along its mechanical cycle of 30 states. The model succeeds in making good correspondence between colloquial molecular biology terms such as “bending,” “stretching,” and twisting with theory. The extensive data in the Results section make this paper one someone wants to study.

Specifically, very long swaths of time must have been spent doing the simulations to collect the very long trajectories from which credible data (and the structural transitions shown in the Results section; approaching seconds). These trajectories are sufficiently long to yield clear energy changes and structure transitions corresponding to steps in nucleotide exchange (Figure 4). These support the hand-over hand model. The paper gains even more credence as it builds on previous takes on the model, from the same laboratory. This is a well thought out, enormous and convincing work. I believe that this model will be a good blueprint that will guide groups. I found it useful that the model is biologically based.

I do not find any obvious flaws. The treatment is rigorous and accessible with some work. Not the least, the manuscript is well written and laid out. The conclusions are supported by the data.

I recommend that this manuscript is accepted for publication.

Response:
We thank the referee very much for the deep appreciation of our manuscript and the detailed letter in its support.
