# Supplementary material for: Elastic analysis bridges structure and dynamics of an AAA+ molecular motor
Source: PLoS Comput Biol. 2025 Oct 24;21(10):e1013596. doi: 10.1371/journal.pcbi.1013596 (PMC12582506; doi:10.1371/journal.pcbi.1013596)
Supplement: Attachment — Submitted filename: PLOSCB-Review.docx [file pcbi.1013596.s017.docx]

Review

9/25/2025

RuvB is an ATPase protein that acts as a motor to drive branch migration of the Holliday junction in bacterial homologous recombination. While branch migration of the Holliday junction is taught in all molecular biology courses, the biophysical basis and exact mechanistic aspects have not been fully worked out. The convergence of the growing collection of well resolved single particle cryoEM structures, and advances in algorithms and GPU computing nodes make this erstwhile problem a good target.

The manuscript, “**Elastic analysis bridges structure and dynamics of an AAA+ molecular motor,**” by VH Mello et al. offers a plausible and accessible model. The authors relate stepwise structural transitions with energy obtained from chemical energy through hydrolysis of ATP to ADP and inorganic phosphate. the **hydrolysis of ATP** (adenosine triphosphate) into ADP (adenosine diphosphate) and an inorganic phosphate, 𝑃𝑖. Structural changes are expressed as elastic deformations quantified as mechanical energy using Hooke’s Law. The energy released is used to push the substrate (DNA) through the protein, RuvB, the helicase. The treatment is sound and consistent.

The model is laid out in the early figures (Figs. 1 and 2), and the results follow in Figure 3 displayed as a per residue plot of a subunit along its mechanical cycle of 30 states. The model succeeds in making good correspondence between colloquial molecular biology terms such as “bending,” “stretching,” and twisting with theory. The extensive data in the Results section make this paper one someone wants to study.

Specifically, very long swaths of time must have been spent doing the simulations to collect the very long trajectories from which credible data (and the structural transitions shown in the Results section; approaching seconds). These trajectories are sufficiently long to yield clear energy changes and structure transitions corresponding to steps in nucleotide exchange (Figure 4). These support the hand-over hand model. The paper gains even more credence as it builds on previous takes on the model, from the same laboratory. This is a well thought out, enormous and convincing work. I believe that this model will be a good blueprint that will guide groups. I found it useful that the model is biologically based.

I do not find any obvious flaws. The treatment is rigorous and accessible with some work. Not the least, the manuscript is well written and laid out. The conclusions are supported by the data.

I recommend that this manuscript is accepted for publication.
